# Effects of enteral immunonutrition enriched with multiple immunonutrients on clinical outcomes of patients who underwent gastric cancer surgery: a systematic review and meta-analysis

**DOI:** 10.3389/fmed.2026.1844823

**Published:** 2026-06-17

**Authors:** Mao Chen, Yan Duan, Yan He, Shan Zhong, Qin Li, Yin Zhao

**Affiliations:** Department of Oncology, The First People’s Hospital of Yibin, Sichuan, China

**Keywords:** clinical outcomes, enteral immunonutrition, enteral nutrition, immune function, infectious complications, inflammatory markers, nutritional status, postoperative gastric cancer

## Abstract

**Objective:**

To evaluate the clinical efficacy of enteral immunonutrition (EIN) compared to standard enteral nutrition (EN) in patients undergoing gastric cancer surgery.

**Methods:**

We searched major databases for randomized controlled trials (RCTs) published up to December 2025. Nineteen RCTs involving 1,653 patients were included. Outcomes assessed included infectious and total complications, length of hospital stay (LOS), gastrointestinal tolerance, and immune-inflammatory markers.

**Results:**

Compared to EN, EIN significantly reduced the incidence of infectious complications (OR = 0.48), gastrointestinal intolerance, and total complications. It also shortened LOS (MD = −1.24 days) and time to first flatus. EIN improved immune parameters (increased IgG, IgM, IgA, transferrin) and reduced inflammatory markers (WBC, CRP). Subgroup analysis indicated that perioperative administration yielded the most significant benefits, while preoperative-only intervention lacked sufficient evidence. Intervention durations > 7 days showed superior outcomes, with regimens within 10 days offering an optimal balance of efficacy and feasibility.

**Conclusion:**

Perioperative enteral immunonutrition, particularly when administered for 7–10 days, is associated with reduced postoperative complications, improved humoral immunity, and shorter hospital stay in patients undergoing gastric cancer surgery. Given the moderate certainty of evidence for primary outcomes, the low certainty for several secondary outcomes, and the evidence of small-study effects for total complications, EIN may be considered as a component of perioperative nutritional support— particularly in patients with established nutritional risk—while routine use should be guided by individualized assessment and confirmed by larger high-quality multicenter trials.

## 1 Introduction

Gastric cancer has a comparatively high incidence and mortality rate and is one of the most prevalent digestive tract cancers. In 2022, data from the World Cancer Research Fund revealed that there were more than 968,000 new cases of gastric cancer globally and approximately 760,000 deaths. The disease was more common among men than among women and has shown trend toward younger age (1, 2). Patients with gastric cancer are often in a hypermetabolic and hypercatabolic state and experience digestive tract absorption disorder, which can lead to malnutrition. Surgery, as the main treatment method for gastric cancer, can worsen malnutrition (3, 4). Malnutrition is not just a side effect; it is an independent risk factor that can aggravate stress response, further weaken the immune system, and disrupt the balance of inflammatory mediators (5). Therefore, such patients generally experience problems such as deterioration of nutritional status, decreased immune function, prolonged operative time, higher incidence of infectious complications, and increased length of hospital stay (6, 7). If the patient’s intestinal condition is suitable, enteral nutrition (EN) support is preferred because it preserves the intestinal ecological environment, promotes intestinal peristalsis, and helps maintain the intestinal mucosal barrier function. EN can also reduce plasma endotoxin levels, help prevent intestinal-derived infection, and is less expensive than parenteral nutrition (PN) (8, 9).

The latest Clinical Nutrition Practice Guidelines for Surgery released by the European Society for Clinical Nutrition and Metabolism (ESPEN) stated that patients who are malnourished undergoing major cancer surgery are among patient populations expected to benefit from perioperative enteral immunonutrition (EIN) (10). EIN refers to adding special nutrients with immunomodulatory effects to EN preparations and formulating various key nutrients in different combinations with added ingredients (11, 12). Frequently utilized immunonutrients encompass arginine, omega-3 (ω-3) polyunsaturated fatty acids, glutamine, nucleotides, ribonucleic acid, cysteine-theanine, probiotics, and dietary fibers. Compared with EN, EIN include immunonutrients, such as ω-3 fatty acids, facilitate the production of proinflammatory eicosanoids, suppress the creation of proinflammatory cytokines, and modulate immune function, resulting in notable anti-inflammatory and immunomodulatory effects. Additionally, arginine is recognized for its ability to boost T-cell activity (13, 14).

These immunonutrients enhance the postoperative immunological response and reduce inflammatory reactions after surgery (15). Glutamine, the primary source of energy for intestinal mucosal cells, promotes mucosal repair and restoration of barrier function (16). Other nutrients that assist immunity, such as dietary fiber, mainly exert immunomodulatory effects by producing short-chain fatty acids through the fermentation of intestinal flora. Fibers can accelerate intestinal peristalsis and shorten the time to first defecation (17). Probiotics can increase the activity of macrophages and lymphocytes, reduce the secretion of tumor necrosis factor-α and interferon-γ, and inhibit intestinal inflammatory reactions (18). The integration of dietary fiber and probiotics with EN can decrease inflammatory responses, reduce diarrhea, and enhance intestinal function recovery, thereby improving immune system after gastric cancer surgery (19–21).

Miyachi et al. (22) believed that immunonutrition preparations containing cysteine and theanine can reduce the inflammatory reactions after gastric cancer surgery. Eicosapentaenoic acid (EPA) supplementation can effectively maintain body weight after esophagectomy (23). However, Ida et al. (24) showed that oral administration of immunonutrition preparations rich in EPA did not significantly improve weight loss in patients undergoing total gastrectomy. Nucleotides, as the basic building units of RNAs, are also substrates for the rapid regeneration of intestinal epithelial cells, promoting villus repair and maintaining intestinal barrier and homeostasis (25). RNAs exert antiviral effects by stimulating the body to produce cytokines such as interferon, promoting cell proliferation and differentiation. As a result, normal functions of rapidly proliferating cells (intestinal epithelial cells and immune cells) require an adequate supply of RNA, and nucleotides can reverse immune deficiencies caused by starvation or specific diets (26). A recent study presented at the North American Surgical Nutrition Summit confirmed that the use of a single-nutrient immunological support program during the perioperative period will not negatively affect clinical outcomes (27). Consequently, specific intervention regimens still exhibit significant variability in efficacy. Giger et al. (28) proposed that preoperative immunonutrition could reduce perioperative inflammation and postoperative complications in patients undergoing abdominal tumor resection. Mochiki et al. (29) revealed that glutamine can promote motor function recovery after gastrectomy, possibly shortening the time to first flatus and the duration postoperative intestinal obstruction. Ruiz-Tovar et al. (30) indicated that preoperative application of ω-3-enriched EIN may reduce postoperative C-reactive protein (CRP) levels and alleviate postoperative pain.

Owing to differences in the patient’s nutritional status, tumor stage, surgical method, immunonutritional components, dosage, administration time, intervention duration, measurement time, etc., systematic evidence on the effect of EIN on postoperative gastric cancer remains inadequately understood. This study aimed to present a more comprehensive and systematic description of the condition. Using the most recent evidence, this study evaluated the effects of EIN on patients with underwent surgery for gastric cancer, focusing on clinical outcome indicators, such as the total postoperative complication rate (TC); infection rate (IC); length of hospital stay (LOS); gastrointestinal intolerance; nutritional status; humoral immunity, cellular immunity, and inflammatory indicators; and time to first flatus and defecation.

## 2 Data and methods

### 2.1 Data

This systematic review and meta-analysis was designed and conducted in accordance with the Preferred Reporting Items for Systematic Reviews and Meta-Analyses guidelines (31). The research process adopted has been registered (number: INPLASY2025120033) and is available on the inplasy.com platform.^1^ No major deviations from the registered protocol occurred during the conduct of this review. The addition of trim-and-fill sensitivity analysis and the formal GRADE certainty-of-evidence assessment described below were introduced *post hoc* as methodological enhancements in response to peer-review recommendations. Eligibility was restricted to English-language reports because the included trial reports rely on detailed numerical outcome reporting (means, standard deviations, event counts) that is difficult to extract reliably across translations, and because pilot screening identified no additional eligible non-English RCT. The potential for language bias is acknowledged in the Limitations.

#### 2.1.1 Inclusion criteria

The population, intervention, comparison, outcome, and study design (PICOS) framework was used, as shown below:

*Participants* (P): Patients were > 18 years old and had undergone gastric cancer surgery.

*Intervention* (I): EIN (included arginine, ω-3 polyunsaturated fatty acids, glutamine, nucleotides, ribonucleic acid, cystine-tyrosine, probiotics, and dietary fiber) used alone or in combination preoperatively, postoperatively, or perioperatively.

*Control* (C): EN, including standard EN.

*Outcome measures* (O): Primary outcome measures included (IC, TC, and LOS. Secondary outcome measures included gastrointestinal intolerance rate, time to first flatus and defecation, nutritional parameters [serum albumin (ALB), transferrin, and prealbumin (PAB)], humoral immune parameters (IgG, IgA, and IgM), cellular immune parameters (CD4+, CD8+, and CD4+CD8+), interleukin-6 (IL-6), CRP, white blood cell (WBC), procalcitonin (PCT), and tumor necrosis factor (TNF-α).

*Study design* (S): Randomized controlled trial (RCT).

#### 2.1.2 Exclusion criteria

The exclusion criteria were as follows: (1) studies not involving EIN and EN in patients with gastric adenocarcinoma or gastrointestinal stromal tumor (GIST) undergoing surgery; (2) literature involving EIN or interventions other than EN; (3) literature comparing outcome indicators without analyzing influencing factors or lacking experimental data; (4) literature with only abstracts, the original text was unavailable, or gray literature; (5) literature comparing the efficacy of EIN with that of EN in patients with non-gastric primary tumors; (6) comprehensive studies on gastric cancer and other malignant tumors that do not separately list individual data results; (7) duplicate literature with suboptimal data reporting or low quality; and (8) exclusion of patients having unresectable tumors, receiving corticosteroids or immunosuppressants, undergoing abdominal radiotherapy, having preoperative infections, or experiencing cardiac, hepatic, or renal insufficiency.

*Language and publication status restrictions*: Only English-language literature was included.

### 2.2 Search strategy

All high-sensitivity search strategies were constructed based on medical subject terms, free words, and Boolean logical operators. A computer was used to search the Cochrane Library, Embase, PubMed, and Web of Science for RCTs from their inception to December 7, 2025. The complete electronic search strategies for PubMed, Embase, Cochrane Library, and Web of Science, including the Boolean structure for each PICOS block, are provided in Supplementary File 1. Reference lists of all included studies and of relevant previous systematic reviews were also hand-searched to identify additional eligible trials.

### 2.3 Literature screening and data extraction

Two researchers (Chen Mao and Zhao Yin) who have received training in evidence-based methodology and basically mastered evidence-based methods independently conducted literature retrieval according to the literature inclusion and exclusion criteria. NoteExpress was used to delete duplicate literature. During literature screening and data extraction, any discrepancies were arbitrated by a third party (Duan Yan). To ensure the reliability and accuracy of the pooled results, studies with missing or incomplete outcomes were excluded. Data extraction was completed using a standardized form in Microsoft Excel. The extracted data included the following: (1) authors, publication date, country, and study type; (2) patient baseline information, surgical type, sample size, intervention measures (EIN formulation), and feeding method; (3) duration of intervention, start and end times; (4) follow-up duration; and (5) outcome indicators and evaluation tools. Title/abstract screening, full-text screening, and data extraction were each conducted independently and in duplicate by two reviewers (CM and ZY); discrepancies were resolved by discussion with a third reviewer (DY). When standard deviations were not reported, they were imputed from standard errors, 95% confidence intervals, or interquartile ranges in accordance with Cochrane Handbook §6.5.2; studies that reported only medians without convertible dispersion measures were excluded from continuous-outcome pooling. No multiple imputation procedures were applied. Outcome definitions were harmonized as follows. Postoperative complications were extracted as reported; where the Clavien–Dindo classification was available, all complications of grade ≥ II were counted as “total complications.” Infectious complications were defined as the composite of surgical site infection, pneumonia, urinary tract infection, intra-abdominal infection or abscess, anastomotic leak with positive culture, line-related infection, and sepsis, consistent with CDC criteria were reported. Gastrointestinal intolerance was defined as any of postoperative diarrhea, abdominal distension or cramping, nausea/vomiting, or feed interruption attributable to gastrointestinal symptoms, as recorded by the original investigators. When a trial reported only a composite event rate without component breakdown, the trial-level definition was retained, and any resulting variability was discussed under sources of heterogeneity. The trial-level operative definitions used for each outcome are summarized in Supplementary Table 2. The certainty of evidence for each outcome was rated using the GRADE approach (GRADEpro GDT) and is summarized in Supplementary Table 3.

### 2.4 Literature quality evaluation

Two researchers independently used the Cochrane risk of bias tool (RoB 2) to evaluate the quality of the included RCTs (32). The following evaluation items were included: random allocation, allocation concealment, blinding for the research subjects and interveners, blinding for the outcome assessors, integrity of outcome data, selective reporting of research results, and other biases. Each item was evaluated as “low risk,” “unclear,” and “high risk” based on the criteria for assessing risk of bias.

### 2.5 Statistical methods

A pooled analysis was conducted using RevMan 5.4 from the Cochrane Collaboration for data evaluation. Various treatment methods were applied across the included studies. Using the Mantel–Haenszel method, risk ratios (RRs) and 95% confidence interval (CI) were calculated for binary variables. Continuous variables were expressed as mean differences (MD) with 95% CI when units and magnitudes for all included studies were consistent (33); otherwise, standardized mean differences (SMDs) were used.

Heterogeneity was assessed using the χ^2^-test along with its *P*-value and the I2 statistic. A random-effects model was utilized if heterogeneity was present among studies (*I* > 50% or *P* < 0.05); otherwise, a fixed-effects model was applied for the pooled analysis. The significance level (α) was established at 0.05; thus, a *P*-value of < 0.05 indicated a significant difference. A subgroup analysis was conducted on the primary outcome indicators based on the intervention time points (perioperative, preoperative, and postoperative) and intervention durations ( ≤ 7 vs. > 7 days; > 10 vs. < 10 days), and subgroup analysis of the intervention timing was performed on the secondary outcome indicators of CD4+ and CD8+ indicators. Where substantial heterogeneity was detected (*I*^2^ > 50%), we conducted leave-one-out analyses to identify studies contributing disproportionately to between-study variance. Studies were considered for exclusion in sensitivity analysis only when they (i) differed clinically from the remainder of the pool in intervention timing, formulation, surgical approach, or outcome definition, or (ii) were identified as statistical outliers by Galbraith plot (standardized residual > |2|). Sensitivity analyses are reported alongside—not as a replacement for—the primary pooled estimates. The full list of studies excluded in each sensitivity analysis, with the corresponding clinical/methodological rationale and the pre- and post-exclusion pooled effects, is provided in Supplementary Table 2. Publication bias was assessed by visual inspection of funnel plots and by Egger’s regression test. For outcomes with statistically significant small-study effects, we additionally applied the Duval and Tweedie trim-and-fill procedure (L_0_ estimator) to obtain an adjusted pooled estimate. Univariable meta-regression on intervention timing and duration was pre-planned but, where fewer than 10 contributing studies were available for an outcome, was not performed because of the limited statistical reliability of meta-regression in small samples (Cochrane Handbook §10.11.4); pre-specified subgroup analyses are presented instead and should be regarded as hypothesis-generating. The certainty of evidence for each outcome was rated using the GRADE approach.

## 3 Results

### 3.1 Study selection and characteristics

**FIGURE 1:**
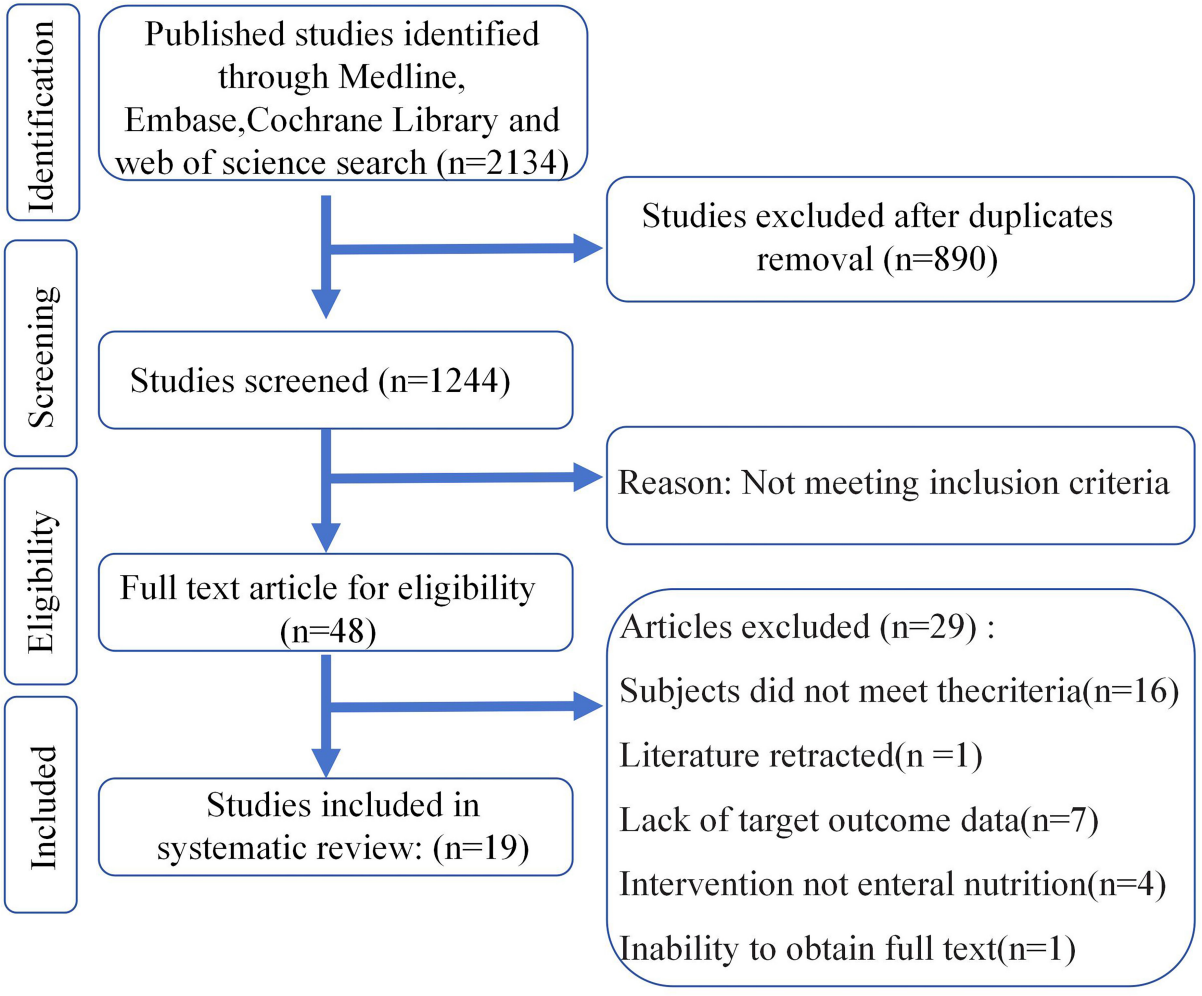
PRISMA flow diagram of study selection. PRISMA flow diagram showing the process of literature identification, screening, eligibility assessment, and inclusion. A total of 2,134 records were identified through database searching. After removing 890 duplicates, 1,244 records were screened based on titles and abstracts, of which 1,196 records were excluded. Subsequently, 48 full-text articles were assessed for eligibility. After full-text review, 29 articles were excluded for the following reasons: ineligible study population (*n* = 16), withdrawn publication (*n* = 1), insufficient outcome data (*n* = 7), non–enteral nutrition intervention (*n* = 4), and unavailable full text (*n* = 1). Ultimately, 19 studies were included in the qualitative synthesis.

A total of 2,134 records were identified through database searches. After removal of duplicates and screening of titles, abstracts, and full texts, 19 RCTs met the inclusion criteria. During full-text assessment, 29 articles were excluded for having ineligible study populations (*n* = 16), insufficient outcome data (*n* = 7), non-EN interventions (*n* = 4), withdrawn publication (*n* = 1), or unavailability of full text (*n* = 1). The study selection process is illustrated in Figure 1.

The included trials comprised 1,653 patients, with 825 assigned to the EIN group and 828 to the EN group. No significant differences in baseline BMI or age were observed between groups. Although substantial heterogeneity was detected in age analysis, this was resolved after exclusion of one study, with no significant between-group difference. Three studies reporting median age were not pooled, which may have introduced bias.

The studies were conducted in China, Italy, Japan, Spain, and Poland and included patients with gastric cancer, including gastric adenocarcinoma and GISTs. Sample sizes ranged from 15 to 127 patients. EIN formulations commonly contained arginine, ω-3 polyunsaturated fatty acids, glutamine, and nucleotides, with additional immunonutrients in some studies. Interventions were administered preoperatively, postoperatively, or throughout the perioperative period, with durations ranging from 5 to 42 days (mean ≈ 11 days). Feeding routes included nasojejunal tube feeding, jejunostomy, oral feeding, and conventional tube feeding. Detailed study characteristics are summarized in Table 1.

**TABLE 1:** Detailed information on the randomized controlled trials (RCTs) included in this study.

Reference, year	Country	Study design	Disease	Study groups(n)	Mean age (year)	Intervention	Therapy	Timing of intervention	Duration (days)	Follow-up	Outcome measures
Xu et al. (19)	China	RCT	Radical resection of gastric cancer	Intervention(*n* = 30)	57.7 ± 9.27	5% GS, probiotics, glutamine, omega-3 fatty acids, nucleotides	Nasogastric tube	17 days postoperatively	7	Postoperative day 2–7	Perioperative nutritional, immune, and inflammatory profiles, as well as postoperative recovery metrics and complications
				Control(*n* = 30)	60.5 ± 8.62	Regular diet					
Yu et al. (40)	China	RCT	Radical resection of gastric cancer	Intervention(*n* = 56)	62.48 ± 10.21	ω3 fatty acids, L-arginine, nucleotides	Oral	Preoperative day 1–7	7	Daily telephone follow-up during hospitalization and for 7 days after discharge until 30 days post-discharge	Infection, nutrition, inflammation, immunity profiles, adverse events, recovery metrics, costs, and short-term mortality.
				Control(*n* = 56)	60.77 ± 10.13	Isotonic, isocaloric standard enteral nutrition					
Ma et al. (43)	China	RCT	Radical resection of gastric cancer	Intervention(*n* = 30)	61.52 ± 9.27	n3 fatty acids, dietary fiber	Oral	4 days before surgery to 1 day before surgery, and 3 days after surgery to 14 days after surgery	16	Postoperative 3.5.7 days, 1 month	Postoperative complications, infection, GI recovery, mucosal barrier, and inflammatory, nutritional, and immune markers
				Control(*n* = 35)	62.51 ± 9.63	Standard enteral nutrition					
Chen et al. (37)	China	RCT	Radical resection of gastric cancer	Intervention(*n* = 20)	59.03 ± 12.55	n3 fatty acids, dietary fiber	Oral	4 days before surgery to 1 day before surgery, and 3 days after surgery to 14 days after surgery	7	Postoperative 3.5.7 days, 1 month	Immunoglobulins (IgA/IgG/IgM), T-cell subsets (CD4/CD8), and inflammatory cytokines (IL-2, IL-6, TNF-α)
				Control(*n* = 20)	Standard enteral nutrition						
Liu et al. (45)	China	RCT	Total gastrectomy for gastric cancer	Intervention(*n* = 28)	57.3 ± 7.1	Glutamine, Arginine and Enteral	Nasogastric tube	Postoperative day 2–7	7	Postoperative days 2, 7, 12	Nutritional and immune profiles, clinical complications, and hospitalization duration (including discharge time and length of stay)
				Control(*n* = 24)	58.4 ± 6.3	Regular diet					
Li et al. (34)	China	RCT	Total/subtotal gastrectomy for gastric cancer	Intervention(*n* = 62)	57.32 ± 10.19	ω3 fatty acids, L-arginine, nucleotides, glutamine	Nasogastric tube	Postoperative day 1–5	5	1 day before surgery and on the 1st, 3rd, and 5th days after surgery	Immune profiles (T-cell subsets, immunoglobulins), inflammatory markers, and serum albumin.prealbumin, and transferrin concentrations.
				Control(*n* = 62)	55.02 ± 9.61	Standard enteral nutrition					
Fujitani et al. (5)	Japan	RCT	Radical resection of gastric cancer	Intervention(*n* = 127)	64 (26–78)	Arginine, nucleotides, 25,26-carboxylic acids	Oral	Preoperative day 1–5	5		Postoperative complications, surgical site infections, and postoperative CRP levels
				Control(*n* = 117)	65 (30–79)	Regular diet					
Ida et al. (24)	Japan	RCT	Gastrectomy	Intervention(n = 63)	65.1(31–79)	Timnodonic Acid	Oral	7 days preoperatively to 21–28 days postoperatively	28		Weight loss, serum albumin, CRP, and surgical complications
				Control(*n* = 60)	65.6(30–80)	Timnodonic Acid					
Marano et al. (35)	Italian	RCT	Major gastrectomy	Intervention(*n* = 54)	66.6 (55–78)	ω3 fatty acids, arginine, nucleotides, glutamine	Jejunostomy tube	6 h postoperatively, 7 days	7	Postoperative day 1,3,7	Infection, complications, ALB/PAB/transferrin, and CD4/CD8/WBC
				Control(*n* = 55)	65.1 (49–83)	Standard enteral nutrition					
Farreras et al. (41)	Spanish	RCT	Gastrectomy	Intervention(*n* = 30)	66.7 ± 8.3	Arginine, omega-3 fatty acids, ribonucleic acid (RNA)	Jejunostomy tube	Postoperative day 1–7	7	4,8 days postoperatively	Hydroxyproline, wound healing, Hb/WBC/Lymphocytes, lb/PAB/TP/Nitrogen balance, LFTs/RFTs, and lipids.
				Control(*n* = 30)	69.2 ± 13.8	Standard enteral nutrition					
Zhao et al. (20)	China	RCT	Distal gastrectomy	Intervention(*n* = 40)	66.52 ± 7.11	Dietary fiber, probiotics and enteral nutrition	Jejunostomy tube	Postoperative day 1–7	7	Postoperative day1,3,5,7	TLC, ALB, PA, TRF, first bowel movement, LOS, intestinal dysfunction, diarrhea
				Control(*n* = 40)	63.53 ± 8.52	EA, enteral nutrition					
Wang et al. (39)	China	RCT	Radical gastrectomy	Intervention(*n* = 65)	60.2 ± 8.7	Arginine, glutamine, β-3 fatty acids	Jejunostomy tube	14 days before the procedure and continuing for 1 month after the procedure	44	Postoperative evaluations were conducted at 1 week, 1 month, and 1 year.	BMI, albumin, PSQI, SAS, SDS, VAS pain, QLQ-C30, complications, LOS, and survival.
				Control(*n* = 65)	58.5 ± 7.9	Standard enteral nutrition					
Xie et al. (21)	China	RCT	Distal gastrectomy	Intervention(*n* = 70)	67.23 ± 10.10	Probiotics and Enteral Nutrition	Jejunostomy tube	6 h postoperatively and continuing until the 8th postoperative day	9	Postoperative day 1–8	IgG, IgA, IgM, IL-6, IL-8, TNF-α, Hb, ALB, PAB, time to distension relief, time to first flatus, LOS, and adverse events
				Control(*n* = 70)	68.36 ± 9.68	EA, enteral nutrition					
Mochiki et al. (29)	Japan	RCT	Distal gastrectomy for gastric cancer	Intervention(*n* = 15)	65 ± 2.6	L-glutamine	Jejunostomy tube	Preoperative day 2–12	11	Postoperative day 1–12	Weight, food intake, first bowel movement, plasma glutamine, and IMC of exercise-complex wave (day 12)
				Control(*n* = 16)	59 ± 2.1	OP, oral placebo					
Ma et al. (38)	China	RCT	Gastric adeno carcinoma or gastric GIST, either selected or undergoing radical surgery	Intervention(*n* = 17)	60.24 ± 12.26	ω3 fatty acids, arginine, glutamine	Nasogastric tube	Preoperative day 3–14	14	from the 3rd preoperative day to the 14th postoperative day or at discharge	ALB, PAB, lipids, glucose, WBC, CRP, IL-6, TNF-α, BUN, Cr, bilirubin, ALT/AST, complications, LOS, and first bowel movement
				Control(*n* = 17)	62.0 ± 9.8	Standard enteral nutrition					
Okamoto et al. (44)	Japan	RCT	Distal/Total Gastrectomy	Intervention(*n* = 30)	66.9 ± 11.5	Arginine, RNA, x-3 polyunsaturated fatty acids	Oral	1 day before surgery; 7 days after surgery	7	Postoperative day 1–7	LOS, body weight, complication duration (infectious/non-infectious/SIRS), and WBC subsets (CD4/CD8/CD16, lymphocytes)
				Control(*n* = 30)	70.9 ± 13.2	Regular diet					
Nakao et al. (17)	Japan	RCT	Distal gastrectomy for gastric cancer	Intervention(*n* = 15)	60.4 ± 7.8	Cystine-threonine	Oral	Preoperative day 4–5	6	Postoperative day 1,4,5,7,14	IL-6, CRP, WBC (NEUI, lymphocytes), REE, body temperature, and ALB.
				Control(*n* = 18)	60.3 ± 5.3	OP, oral placebo					
Scislo et al. (36)	Poland	RCT	Total gastrectomy	Intervention(*n* = 44)	62.6 ± 11.9	Arginine, glutamine, nucleotides, β-3 fatty acids	Nasogastric tube	Postoperative care should be initiated 8–16 h after surgery and continued until the 6th postoperative day.	7	6 months and 1 year postoperatively	IL-6, CRP, WBC (NEUI, lymphocytes), REE, body temperature, and ALB.
				Control(*n* = 54)	62.9 ± 10.7	Standard enteral nutrition					
Zheng et al. (42)	China	RCT	Radical gastrectomy for gastric cancer	Intervention(*n* = 29)	57.82 ± 12.79	ω-3 fatty acids, arginine, nucleotides	Oral	From day 3 preoperatively to day 14 postoperatively	15	3 days preoperatively; postoperatively, 3, 7, 14	Perioperative serum parameters (WBC, lymphocytes, CD3/4/8, IgG, CRP, ALB, PAB, TP, Hb), intestinal barrier markers (DAO, D-lactate, endotoxin), fecal metagenomic sequencing, and postoperative complications.
				Control(*n* = 29)	59.207 ± 10.31	Standard enteral nutrition					

RCT, randomized controlled trial; ALB, albumin; PAB, prealbumin; CRP, C-reactive protein; IL-6, interleukin-6; TNF-α, tumor necrosis factor-α; EN, enteral nutrition; ω-3 FA, omega-3 fatty acids; GIST, gastrointestinal stromal tumor.

### 3.2 Quality evaluation results

The risk of bias of the included studies was assessed using the Cochrane RoB 2 tool, which evaluates seven methodological domains. Overall, 14 studies reported adequate randomization methods and were judged as low risk, whereas the remainder were assessed as having moderate risk. Allocation concealment was rated as low risk in seven studies and moderate risk in the others. Blinding was adequately performed in nine studies (low risk), whereas three studies without blinding were rated as high risk. The remaining studies were judged as having moderate risk. Outcome assessment blinding was implemented in 16 studies and rated as low risk. Most studies demonstrated low risk of selective reporting, with the rest assessed as moderate risk. One study was rated as high risk for other sources of bias, nine as low risk, and the rest as moderate risk. Detailed results are presented in Table 2.

**TABLE 2:** Risk of bias assessment of the included studies using the Cochrane RoB 2 tool.

References	Random sequence generation (selection bias)	Allocation concealment (selection bias)	Blinding of participants and personnel (performance bias)	Blinding of outcome assessment (detection bias)	Incomplete outcome data (attrition bias)	Selective reporting (reporting bias)	Other bias
Xu et al. (19)	Random digit	Unknown	Unknown	Low risk	Complete outcome data	Low risk	Low risk
Yu et al. (40)	Computer random	Unknown	High risk	Low risk	Complete outcome data	Low risk	Low risk
Ma et al. (43)	Unknown	Sealed opaque envelopes	Single-blind	Low risk	Complete outcome data	Low risk	Low risk
Chen et al. (37)	Random digit	Unknown	Unknown	Low risk	Complete outcome data	Unknown	Unknown
Liu et al. (45)	Unknown	Unknown	Unknown	Low risk	Complete outcome data	Low risk	High risk
Li et al. (34)	Computer random	Low Risk	Double-blind	Low risk	Complete outcome data	Low risk	Low risk
Fujitani et al. (5)	Computer random	Unknown	Unknown	Unknown	Complete outcome data	Low risk	Low risk
Ida et al. (24)	Computer random	Unknown	Low risk	Low risk	Complete outcome data	Low risk	Low risk
Marano et al. (35)	Unknown	Unknown	Unknown	Low risk	Complete outcome data	Low risk	Unknown
Farreras et al. (41)	Random digit	Unknown	Double-blind	Low risk	Complete outcome data	Low risk	Unknown
Zhao et al. (20)	Random sequence generation	Sealed opaque envelopes	Low risk	Low risk	Complete outcome data	Low risk	Unknown
Wang et al. (39)	Computer random	Sealed opaque envelopes	High risk	Low risk	Complete outcome data	Low risk	Low risk
Xie et al. (21)	Unknown	Unknown	Unknown	Low risk	Complete outcome data	Low risk	Unknown
Mochiki et al. (29)	Random digit	Unknown	Single-blind	Unknown	Complete outcome data	Low risk	Low risk
Ma et al. (38)	Random sequence generation	Sealed opaque envelopes	Double-blind三盲	Low risk	Complete outcome data	Low risk	Unknown
Okamoto et al. (44)	Random digit	Sealed opaque envelopes	Low risk	Low risk	Complete outcome data	Low risk	Low risk
Nakao et al. (17)	Block randomization method	Sealed opaque envelopes	Single-blind	Low risk	Complete outcome data	Low risk	Low risk
Scislo et al. (36)	Computer random	Unknown	High risk	Low risk	Complete outcome data	Low risk	Low risk
Zheng et al. (42)	Unknown	Unknown	Unknown	Low risk	Complete outcome data	Low risk	Low risk

### 3.3 Intervention characteristics

This review summarized the intervention strategies of 19 studies (Table 3). Most studies (15/19) used multi-component immunonutrition, most commonly combinations of arginine, ω-3 fatty acids, glutamine, and nucleotides, and the combinations of ω-3 fatty acids + arginine + glutamine ± nucleotides were the most frequently reported (34–39). Only four studies used a single immunonutrient. Control groups mainly received EN or standard EN (SEN), whereas a few used oral placebo or normal diet. EIN was administered preoperatively only (3 studies) (5, 40, 41), both preoperatively and postoperatively (6 studies) (24, 38, 39, 42–44), or postoperatively only (10 studies) (19–21, 29, 34–37, 41, 45). Outcome assessment primarily focused on infection-related complications, LOS, immune function, and inflammatory markers. To facilitate cross-study comparison, the included EIN regimens were grouped into five categories on the basis of their dominant immunonutrient profile: (i) arginine-based formulations (typically combined with nucleotides and/or ω-3 fatty acids); (ii) ω-3 fatty-acid (EPA/DHA)-dominant formulations; (iii) glutamine-based formulations; (iv) probiotic and/or dietary-fiber-based formulations; and (v) cysteine/theanine-based formulations. Daily doses of the major components were extracted where reported and are summarized in Table 1; the categorical distribution is shown in Table 3. The biological rationale of these categories differs substantially (immune-modulating amino acids, anti-inflammatory polyunsaturated fatty acids, gut-mucosal substrates, and microbiota modulators), which provides a mechanistic basis for the between-study heterogeneity observed in several pooled analyses and motivates the cautious interpretation of pooled effects as a regimen-averaged estimate rather than as the effect of any single nutrient.

**TABLE 3:** Summary of enteral immunonutrition intervention strategies in the included studies.

Category	Description
Type of immunonutrient intervention	Multi-component immunonutrition was used in the majority of studies (15/19), whereas single immunonutrient supplementation was reported in 4 studies
Most common immunonutrient combinations	Arginine + ω-3 fatty acids + glutamine + nucleotides (6 studies); Arginine + ω-3 fatty acids + glutamine (6 studies)
Other reported immunonutrients	RNA, nucleotides, probiotics, dietary fiber, eicosapentaenoic acid, and other nutrients
Single immunonutrient interventions	Used in 4 studies
Control group interventions	Enteral nutrition (EN) or standard enteral nutrition (SEN) in most studies; oral placebo or normal diet in a minority of studies
Timing of EIN intervention	Preoperative only: 3 studies; Pre- and postoperative: 6 studies; Postoperative only: 10 studies
Intervention duration	Variable across studies (ranging from short perioperative courses to extended postoperative support)
Primary outcome domains evaluated	Infection-related complications, length of hospital stay, immune function, and inflammatory markers

### 3.4 Meta-analysis results

#### 3.4.1 Primary outcomes

**FIGURE 2:**
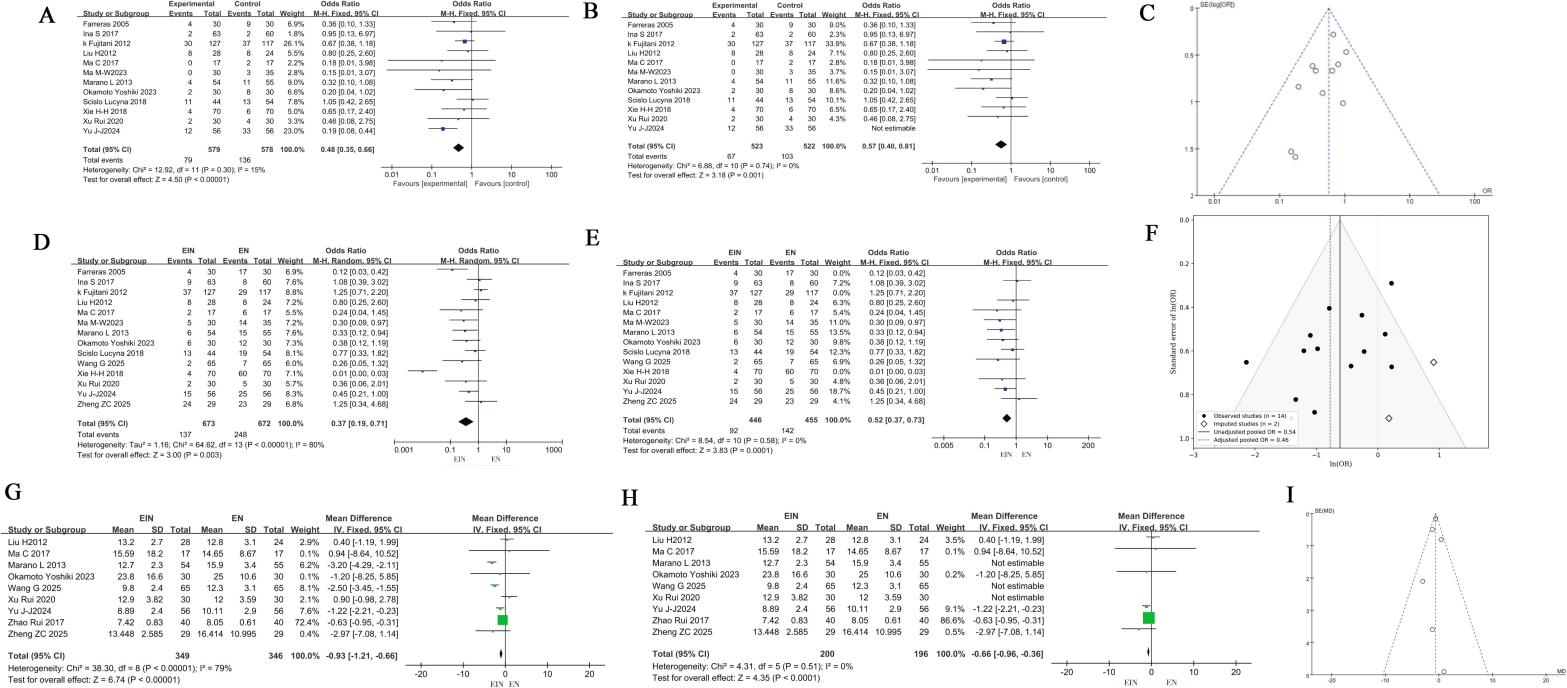
Effect of enteral immunonutrition on postoperative infectious complications, total complication rate and length of hospital stay (LOS). **(A)** Forest plot of the primary meta-analysis including 12 randomized controlled trials (1,157 patients), showing a significantly lower incidence of IC in the EIN group than in the EN group (OR = 0.48, 95% CI 0.35–0.66), with low heterogeneity (*I*^2^ = 15%). **(B)** Forest plot of sensitivity analysis after exclusion of one study, demonstrating consistent results with no heterogeneity (*I*^2^ = 0%; OR = 0.57, 95% CI 0.40–0.81). **(C)** Funnel plot assessing publication bias, indicating minimal asymmetry. **(D)** Forest plot of the primary meta-analysis including 14 randomized controlled trials (1,345 patients), showing a significantly lower TC in the EIN group than in the EN group (OR = 0.37, 95% CI 0.19–0.71), with substantial heterogeneity (*I*^2^ = 80%). **(E)** Forest plot of sensitivity analysis after exclusion of three studies, demonstrating consistent results with no heterogeneity (*I*^2^ = 0%; OR = 0.52, 95% CI 0.37–0.73). **(F)** Funnel plot evaluating publication bias, indicating minimal asymmetry. A trim-and-fill (Duval and Tweedie, L_0_) adjustment for total complications has been added in this revised version; imputed studies and the adjusted pooled estimate are overlaid on the funnel plot, with numerical output reported in Supplementary Table 1. **(G)** Forest plot of the primary meta-analysis including nine randomized controlled trials (695 patients), showing a significantly shorter LOS in the EIN group than in the EN group (MD = - 1.24 days, 95% CI - 2.22 to - 0.25), with substantial heterogeneity (*I*^2^ = 79%). **(H)** Forest plot of sensitivity analysis after exclusion of three studies, demonstrating consistent results with no heterogeneity (*I*^2^ = 0%; MD = - 0.66 days, 95% CI - 0.96 to - 0.36). **(I)** Funnel plot assessing publication bias, indicating minimal asymmetry.

IC: Twelve RCTs involving 1,157 patients reported infectious complications. Low heterogeneity was observed (*I*^2^ = 15%), and a fixed-effect model showed a significantly lower incidence of IC in the EIN group than in the EN group (13.6% vs. 23.5%; OR = 0.48, 95% CI 0.35–0.66, *P* < 0.00001) (Figure 2A). Sensitivity analysis, excluding one study, yielded consistent results with no heterogeneity (*I*^2^ = 0%; OR = 0.57, 95% CI 0.40–0.81, *P* = 0.001). Publication bias was minimal, and the certainty of evidence was moderate (Figures 2B,C).

TC: Fourteen RCTs including 1,345 patients reported total complications. A random-effects model revealed a significantly reduced TC in the EIN group compared with the EN group (20.4% vs. 36.9%; OR = 0.37, 95% CI 0.19–0.71, *P* = 0.003), despite substantial heterogeneity (*I*^2^ = 80%) (Figure 2D). The exclusion of three studies eliminated heterogeneity (*I*^2^ = 0%), and the effect remained significant (OR = 0.52, 95% CI 0.37–0.73, *P* = 0.0001). Publication bias was low, and the certainty of evidence was moderate (Figures 2E,F).

LOS: Nine trials comprising 695 patients reported LOS. EIN significantly shortened LOS compared with EN (MD = −1.24 days, 95% CI −2.22 to −0.25, *P* = 0.01), with substantial heterogeneity (*I*^2^ = 79%) (Figure 2G). After removing three studies, heterogeneity was resolved (*I*^2^ = 0%), and the reduction in LOS remained significant (MD = −0.66 days, 95% CI −0.96 to −0.36, *P* < 0.0001). Funnel plots suggested minimal publication bias, and the certainty of evidence was moderate (Figures 2H,I).

#### 3.4.2 Secondary outcomes

##### 3.4.2.1 Gastrointestinal recovery and postoperative adverse events

**FIGURE 3:**
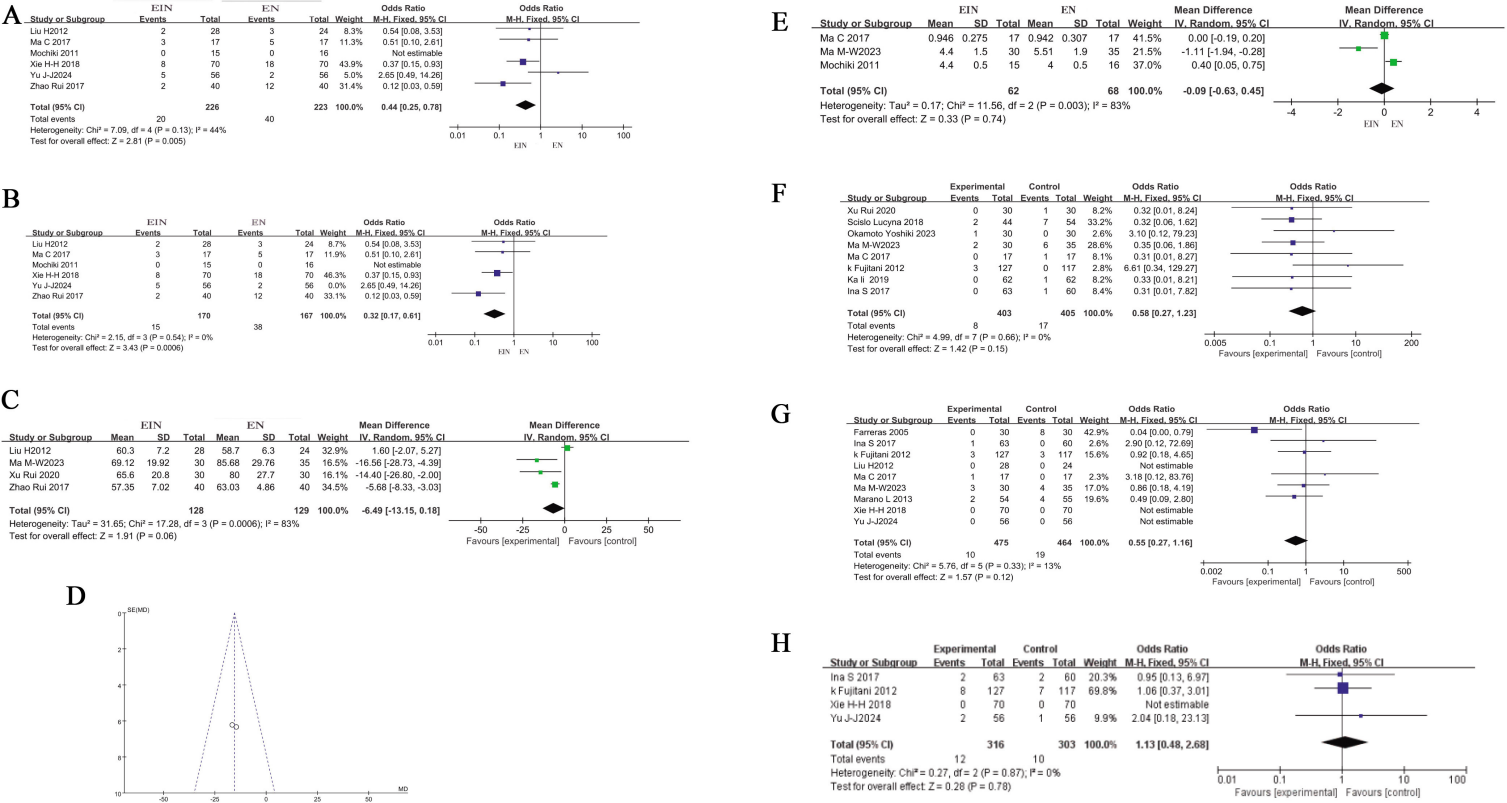
Effects of enteral immunonutrition on gastrointestinal recovery outcomes and additional postoperative outcomes. **(A)** Forest plot of the incidence of gastrointestinal intolerance, showing a significantly lower rate in the EIN group compared with the EN group. **(B)** Forest plot of time to first flatus, indicating a shorter time to gastrointestinal function recovery in patients receiving EIN. **(C)** Funnel plot assessing publication bias for gastrointestinal recovery outcomes. **(D)** Time to first defecation. **(E)** Postoperative bleeding. **(F)** Postoperative anastomotic leakage. **(G)** Postoperative pancreatic fistula. **(H)** Postoperative pancreatic fistula.

EIN was associated with a significantly lower incidence of gastrointestinal intolerance compared with EN across six RCTs involving 418 patients (OR = 0.44, 95% CI 0.25–0.78) (Figure 3A). This effect remained robust after sensitivity analysis, with no heterogeneity and minimal publication bias, and the certainty of evidence was rated as moderate (Figure 3B). Significant heterogeneity was observed across four trials reporting the time to first flatus (257 patients; MD = −6.49), and the pooled analysis using a random-effects model showed no significant difference between groups. The exclusion of two heterogeneous studies eliminated heterogeneity (*I*^2^ = 0%, *P* = 0.81). However, due to potential publication bias, the results should be interpreted with caution (Figures 3C,D).

In contrast, no significant differences in the time to first defecation, postoperative bleeding, anastomotic leakage, or postoperative pancreatic fistula were observed between the EIN and EN groups (Figures 3E–H). These outcomes were generally characterized by high heterogeneity, limited sample sizes, or inconsistent results across studies. Funnel plot asymmetry and sensitivity analyses further suggested potential publication bias, and the overall certainty of evidence for these outcomes ranged from low to very low. Mortality. Among the 19 included trials, only three reported mortality-related outcomes within trial-specific follow-up windows: Yu et al. (40) reported 1-month all-cause mortality, Scislo et al. (36) reported 6-month and 1-year survival, and Wang and Pan (39) reported 1-year survival. Event counts were sparse (single-digit deaths in most arms), follow-up windows were heterogeneous (1 month to 1 year), and cause-of-death adjudication was inconsistent. No individual trial reported a statistically significant between-group difference in mortality, and quantitative pooling was therefore not undertaken because it would have produced an unstable and clinically misleading estimate. This pattern is consistent with previous systematic reviews of perioperative immunonutrition in upper-gastrointestinal cancer surgery, which similarly reported no significant mortality benefit (46, 47). The insufficient and inconsistent reporting of mortality across trials is recognized as a key limitation of the current evidence base (see Limitations).

##### 3.4.2.2 Humoral immunity indicators

**FIGURE 4:**
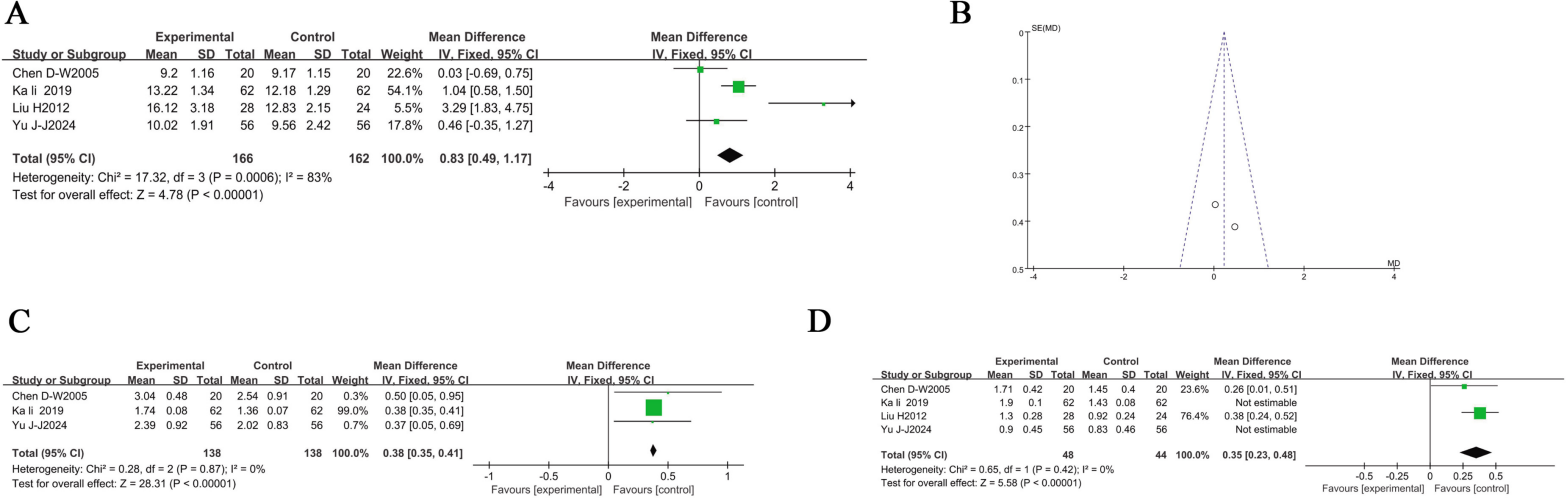
Effects of enteral immunonutrition on humoral immunity indicators. **(A)** Forest plot of IgG concentrations, showing a small increase in the EIN group that was not sustained after sensitivity analysis. **(B)** Funnel plot assessing publication bias for IgG. **(C)** Forest plot of IgA concentrations, demonstrating a significant increase in the EIN group compared with EN. **(D)** Forest plot of IgM concentrations, showing a significant and robust increase in the EIN group that remained consistent after sensitivity analysis.

Four RCTs involving 328 patients evaluated IgG concentrations. Although the pooled analysis showed a small increase in the EIN group (MD = 1.02, *P* = 0.03), this effect was not sustained after sensitivity analysis, suggesting the lack of stable difference between the EIN and EN groups (Figures 4A,B). Three trials including 276 patients reported IgA, demonstrating a significant increase in the EIN group compared with the EN group (MD = 0.38, *P* < 0.00001) (Figure 4C). For IgM, four RCTs (328 patients) showed a significant and consistent increase with EIN (MD = 0.31, *P* = 0.001), which remained robust after sensitivity analysis (*P* < 0.00001). Publication bias was minimal, and the certainty of evidence was rated as moderate for all outcomes (Figure 4D).

##### 3.4.2.3 Nutritional biochemical test indicators

**FIGURE 5:**
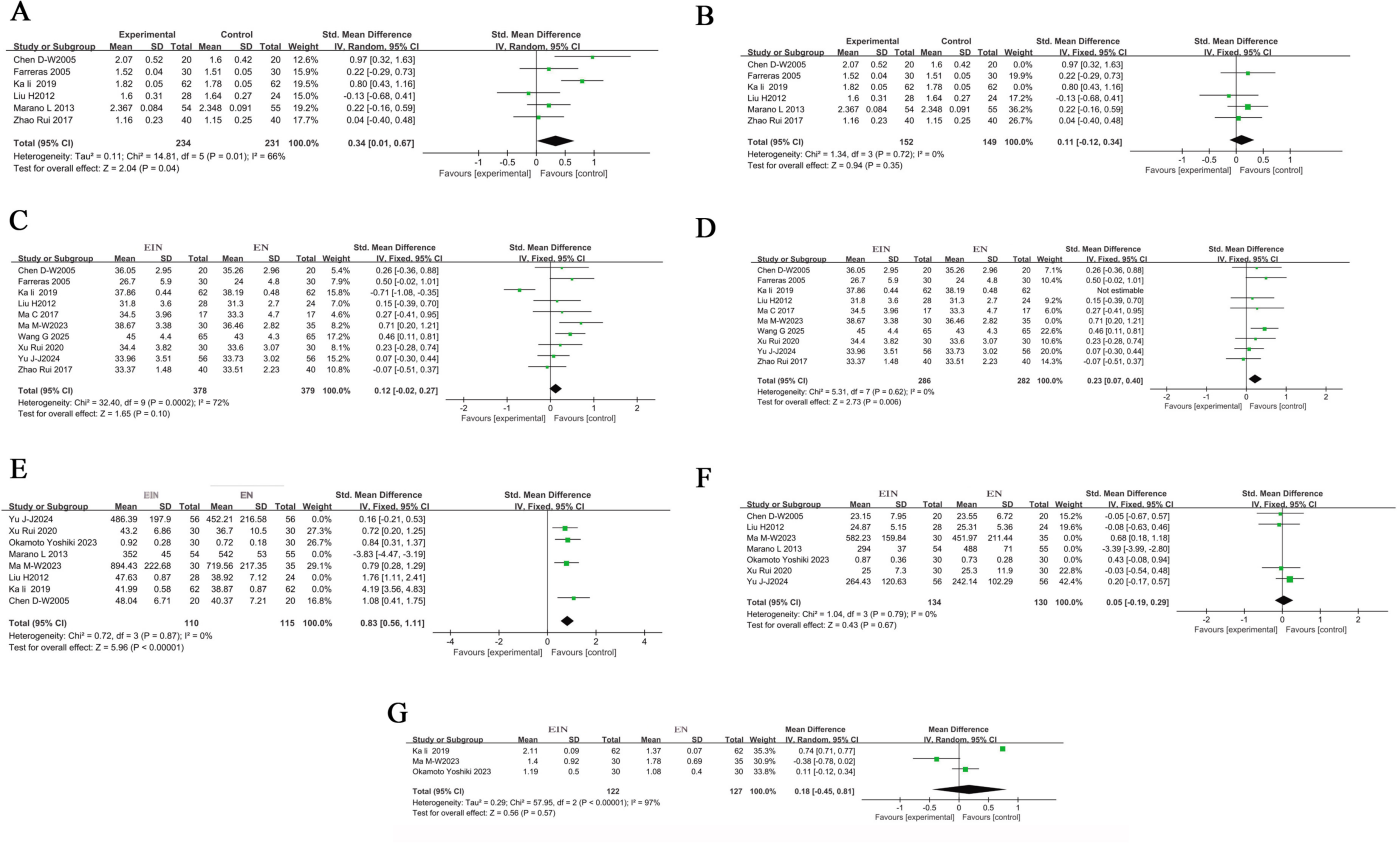
Effects of enteral immunonutrition on nutritional biochemical indicators and cellular immune indicators. **(A)** Forest plot of transferrin (TRF), showing a small but statistically significant increase in the EIN group in the primary analysis. **(B)** Sensitivity analysis for TRF, demonstrating that the initial effect was not maintained after exclusion of heterogeneous studies. **(C)** Forest plot of albumin (ALB), showing no significant difference between the EIN and EN groups in the primary analysis. **(D)** Sensitivity analysis for ALB, indicating a modest increase favoring EIN after exclusion of heterogeneous studies. **(E)** Forest plot of CD4^+^ levels, showing a significant increase in the EIN group following sensitivity analysis. **(F)** Forest plot of CD8^+^ levels, demonstrating no significant difference between the EIN and EN groups. **(G)** Forest plot of the CD4^+^/CD8^+^ ratio, showing no significant between-group difference.

**FIGURE 6:**
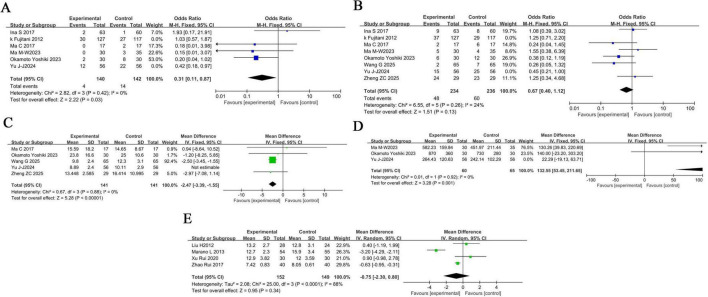
Subgroup analyses according to duration of enteral immunonutrition. **(A)** Total complication rate (TC) in patients receiving EIN for > 7 days, showing a significant reduction. **(B)** Length of hospital stay in patients receiving EIN for > 7 days, demonstrating a significant shortening of hospitalization. **(C)** Incidence of infectious complications (IC) in patients receiving EIN for ≤ 7 days, showing a significant reduction. **(D)** Total complication rate (TC) in patients receiving EIN for ≤ 7 days, showing a significant reduction. **(E)** Length of hospital stay in patients receiving EIN for ≤ 7 days, showing no significant difference between groups.

For nutritional biochemical indicators, pooled analyses showed a small but significant increase in transferrin (TRF) with EIN compared with EN (SMD = 0.34, *P* = 0.04); however, this difference disappeared after sensitivity analysis (SMD = 0.11, *P* = 0.35), indicating an unstable effect (Figures 5A,B). Similarly, no significant difference in ALB levels was observed in the primary analysis (SMD = 0.17, *P* = 0.24), whereas sensitivity analysis revealed a modest increase favoring EIN (SMD = 0.23, *P* = 0.006), suggesting a potential but inconsistent benefit (Figures 6C,D). For prealbumin (PAB), the initial pooled result showed a significant increase with EIN (SMD = 0.30, *P* = 0.0004); however, this advantage was not maintained after exclusion of heterogeneous studies (SMD = −0.05, *P* = 0.62) (Supplementary Figures 1A,B).

Regarding cellular immune indicators, no significant differences in the primary analyses of CD4^+^, CD8^+^, or the CD4^+^/CD8^+^ ratio were observed between the groups. Sensitivity analysis suggested a significant increase in CD4^+^ levels after exclusion of heterogeneous studies (SMD = 0.83, *P* < 0.00001), whereas CD8^+^ levels and the CD4^+^/CD8^+^ ratio remained non-significant in all analyses (Figures 5C,D,F). Overall, these findings indicate that the effects of EIN on nutritional biochemical and cellular immune markers are inconsistent and sensitive to study heterogeneity, with low to moderate certainty of evidence, and should be interpreted cautiously.

Other nutritional parameters. Beyond ALB, PAB, and TRF, several individual trials reported supplementary nutritional indicators, but these were not amenable to quantitative pooling because they were reported in fewer than three studies in compatible units and at inconsistent measurement time points. Postoperative body weight or BMI change was reported in Ida et al. (24), Ma et al. (38), Wang and Pan (39), and Yu et al. (40), with no consistent between-group difference. Hemoglobin recovery was reported in Liu et al. (45), Xie et al. (21), Zhao et al. (20), and Zheng et al. (42), with no significant difference between EIN and EN. Total lymphocyte count was reported in Farreras et al. (41), Marano et al. (35), Nakao et al. (17), and Zhao et al. (20), without a consistent pattern. Nitrogen balance was reported only by Farreras et al. (41). ALB, PAB, and TRF reflect only the visceral protein pool and are confounded by the postoperative acute-phase response; functional or composite measures such as handgrip strength, PG-SGA, or the GLIM criteria were not assessed in any of the included trials. This limited and inconsistent nutritional reporting is therefore acknowledged as a constraint on the strength of the conclusions and is addressed in the Limitations.

##### 3.4.2.4 Inflammatory markers

**FIGURE 7:**
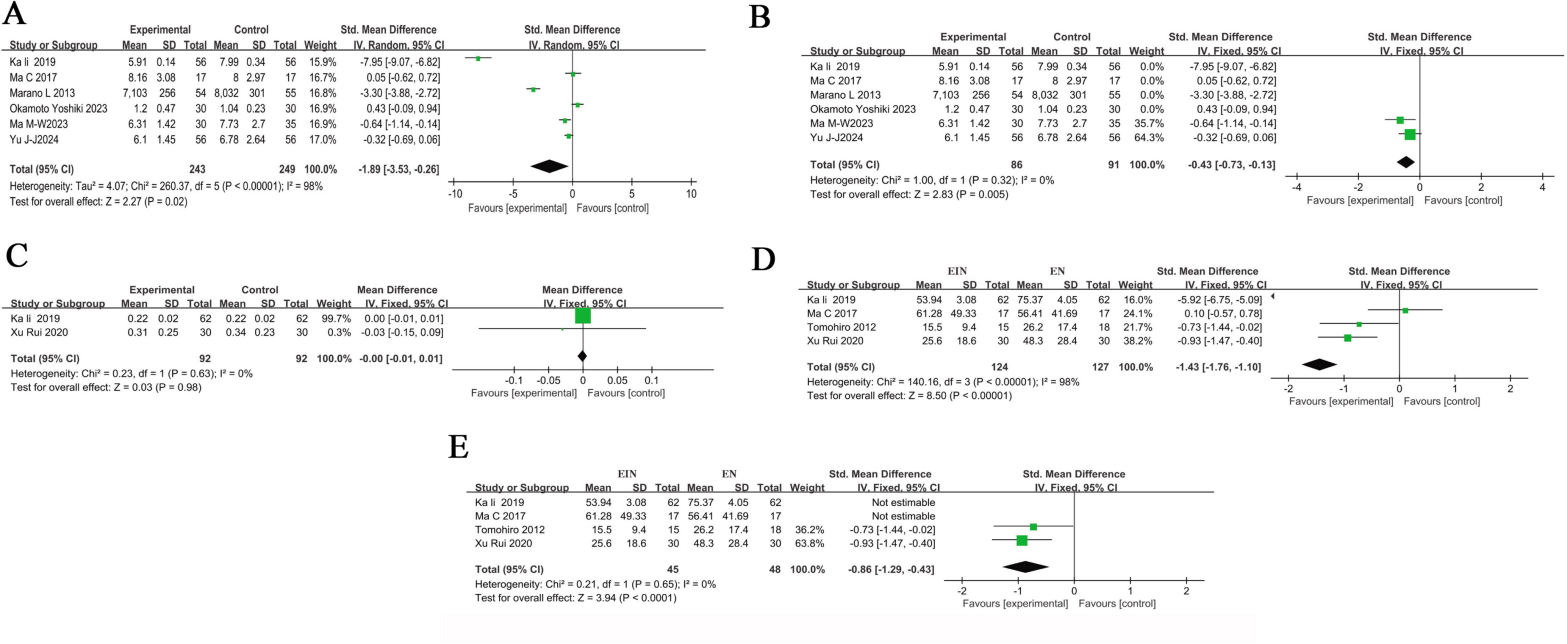
Effects of enteral immunonutrition on inflammatory and infection-related biomarkers. **(A)** Forest plot of white blood cell count (WBC) from six randomized controlled trials (492 patients), showing a significant reduction in the EIN group. **(B)** Forest plot of sensitivity analysis for WBC after exclusion of heterogeneous studies, demonstrating a consistent effect. **(C)** Forest plot of procalcitonin (PCT) from two randomized controlled trials (184 patients), showing no significant difference between the EIN and EN groups. **(D)** Forest plot of C-reactive protein (CRP) from four randomized controlled trials (251 patients), indicating a significant reduction in the EIN group. **(E)** Forest plot of sensitivity analysis for CRP, confirming the robustness of the association.

For WBC, six RCTs including 492 patients showed a significant reduction in the EIN group compared with the EN group in the pooled analysis (SMD = −0.91, *P* < 0.00001). This effect remained significant after sensitivity analysis excluding heterogeneous studies (SMD = −0.43, *P* = 0.005), suggesting a potential reduction in postoperative inflammatory response, although publication bias and low certainty of evidence warrant cautious interpretation (Figures 7A,B). Regarding PCT, two RCTs involving 184 patients demonstrated no significant difference between the EIN and EN groups (*P* = 0.98). The limited sample size and potential publication bias reduced confidence in this finding (Figure 7C). For CRP, four RCTs (251 patients) showed a significant reduction in the EIN group in the primary analysis (SMD = −1.43, *P* < 0.00001). This association remained significant after sensitivity analysis (SMD = −0.86, *P* < 0.0001), indicating a consistent trend toward lower CRP levels with EIN; however, evidence certainty was low because of heterogeneity and publication bias (Figures 7D,E).

In contrast, IL-6 showed no significant difference in the primary analysis (*P* = 0.74). Sensitivity analysis suggested a reduction favoring EIN (SMD = −0.80, *P* = 0.001); however, the small sample size and instability of results require cautious interpretation of this finding (Supplementary Figures 1C,D). Similarly, pooled analyses of TNF-α across three RCTs (184 patients) showed no significant difference between the groups (*P* = 0.19), with substantial heterogeneity and low certainty of evidence (Supplementary Figure 2E).

Overall, EIN exerted a potential anti-inflammatory effect, particularly reflected by reductions in WBC and CRP, whereas effects on PCT, IL-6, and TNF-α were inconsistent and inconclusive, with the overall certainty of evidence rated as low.

##### 3.4.2.5 Subgroup analysis

**FIGURE 8:**
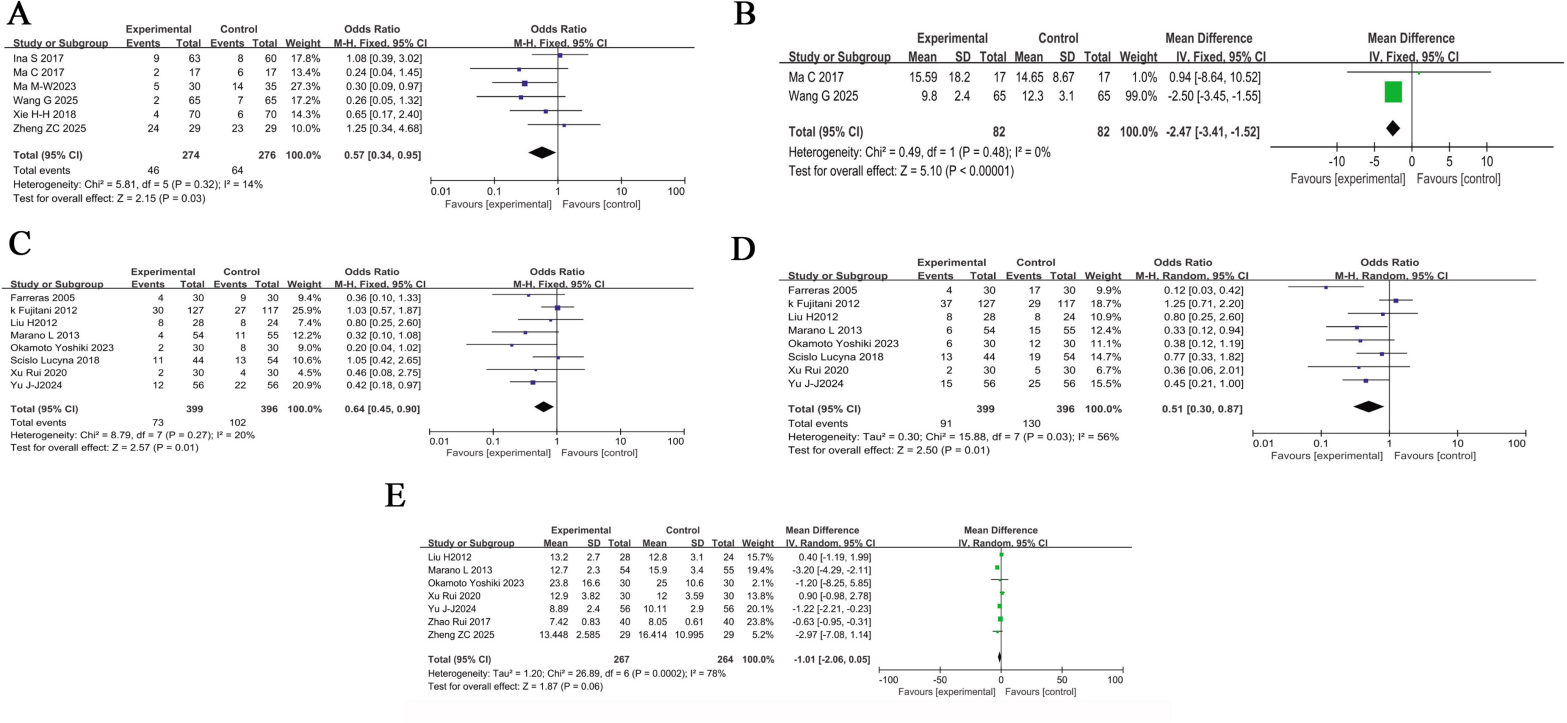
Subgroup analyses according to intervention timing and duration. **(A)** Incidence of infectious complications (IC) stratified by intervention timing. **(B)** Total complication rate (TC) stratified by intervention timing. **(C)** Length of hospital stay stratified by intervention timing. **(D)** CD8^+^ levels stratified by intervention timing, showing a significant increase with perioperative EIN. **(E)** CD4^+^ levels stratified by intervention timing, showing no significant difference between groups.

Subgroup analysis of the primary outcome measures was conducted according to the intervention timing (postoperative, perioperative, and preoperative) and intervention duration (>7 and ≤ 7 days, > 10 and ≤ 10 days), and subgroup analysis of the intervention timing was performed for the secondary outcome measures of CD4+ and CD8+ indices. Subgroup analyses showed that the timing and duration of EIN substantially affected clinical outcomes. As regards intervention timing, perioperative EIN was associated with the most consistent benefits, including a reduced incidence of IC (OR = 0.31, *P* = 0.03) and TC (OR = 0.60, *P* = 0.02), as well as a significantly shorter LOS (MD = −2.47 days, *P* < 0.00001) (Figures 8A–C). Perioperative EIN also significantly improved CD8^+^ levels (P = 0.001), whereas no significant effect was observed on CD4^+^ levels (Figures 8D,E). Postoperative EIN significantly reduced IC (OR = 0.59, *P* = 0.04) and TC (OR = 0.45, *P* = 0.0007) and increased CD4^+^ levels (*P* = 0.01), but showed no significant effect on hospital stay or CD8^+^ levels (Supplementary Figures 2A–E). Evidence for preoperative EIN alone was limited, with no significant differences observed. The preoperative subgroup only included three RCTs, and several-duration subgroups also included a small number of trials. Accordingly, these subgroup estimates should be interpreted as hypothesis-generating rather than confirmatory.

As regards intervention duration, EIN administered for > 7 days significantly reduced TC (OR = 0.57, *P* = 0.03) and shortened the LOS (MD = −2.47 days, *P* < 0.00001) (Figures 6A,B), whereas EIN for ≤ 7 days significantly reduced both IC (OR = 0.54, *P* = 0.01) and TC (OR = 0.51, *P* = 0.01) but did not significantly shorten the LOS (Figures 6C–E). Furthermore, EIN for > 10 days significantly reduced TC (OR = 0.56, *P* = 0.04) and shortened the LOS (MD = −2.49 days, *P* < 0.00001) (Supplementary Figures 3A,B), whereas EIN for ≤ 10 days significantly reduced IC (OR = 0.64, *P* = 0.008) and TC (OR = 0.53, *P* = 0.009) without a significant effect on LOS (Supplementary Figures 3C–E).

##### 3.4.2.6 Publication bias

Egger regression analysis revealed that a total of 14 studies were included for the analysis of overall complications. The results indicated a significant small sample effect (intercept = −2.38, 95% CI −4.35, −0.41; *P* = 0.022) and publication bias. For infection-related complications, 12 studies were included (intercept = −1.20, 95% CI −2.54, 0.13; *P* = 0.072), suggesting that the possibility of small sample effect or publication bias could not be entirely ruled out. The tau^2^ value of 0.0039 indicated low heterogeneity among studies. However, no publication bias was identified for the outcome measure of ALB level (intercept = −0.07, 95% CI −4.06 to 3.83, *P* = 0.949) (Supplementary Table 1). Given the statistically significant small-study effect detected for total complications, the Duval and Tweedie trim-and-fill procedure was additionally applied to this outcome. The procedure imputed missing studies on the right-hand side of the funnel, and the adjusted pooled estimate remained statistically significant although attenuated relative to the unadjusted effect. The funnel plot incorporating the trim-and-fill adjustment is presented in Figure 2F, and the corresponding numerical results are reported alongside the original Egger output in the revised Supplementary Table 1. The persistence of a clinically meaningful effect after adjustment suggests that, while small-study effects probably contributed to inflation of the unadjusted pooled estimate, the overall protective association is unlikely to be entirely an artifact of publication bias. This caveat is reflected both in the strength of the conclusions and in the Limitations.

## 4 Discussion

Globally, gastric cancer remains the fifth most common malignancy and the fourth leading cause of cancer-related death (48). Postoperative recovery in patients with gastric cancer is often influenced by surgical stress, inflammatory responses, and immune dysfunction (3). Nutritional support plays an important role in perioperative management and is mainly delivered through EN or PN. Although PN can provide adequate nutritional supplementation, it may also increase the risk of mechanical complications, infections, and metabolic disorders. Some studies have suggested that PN enriched with ω-3 fatty acids may reduce infection risk (49), whereas others reported that intravenous immunonutrition might be associated with higher rates of sepsis and postoperative infections in patients with gastric cancer (50, 51). In contrast, previous systematic analyses have indicated that EIN can improve the nutritional status of patients with upper gastrointestinal cancer (47).

Several meta-analyses have explored the potential benefits of EIN. Previous studies have reported that EIN could shorten hospital stay and reduce postoperative complications (52, 53), although excessively prolonged intervention might increase infection risk (54). Other analyses suggested that EIN improves certain immune and nutritional indicators, such as CD8^+^ cells, immunoglobulins, and PAB levels (55), whereas supplementation with specific components such as ω-3 fatty acids increase CD4^+^ levels and the CD4^+^/CD8^+^ ratio (56, 57). Nevertheless, the overall clinical benefits of EIN compared with standard EN remain controversial, particularly regarding outcomes such as LOS, inflammatory markers, and nutritional indicators.

### 4.1 Clinical outcomes

This meta-analysis demonstrated that EIN provides clear clinical benefits for patients undergoing surgery for gastric cancer. EIN significantly reduced IC (OR = 0.48, *P* < 0.00001) and TC (OR = 0.37, *P* = 0.003) and shortened LOS (MD = −1.24 days, *P* = 0.01), with stable sensitivity analyses and moderate certainty of evidence. These findings are consistent with previous studies showing that immunomodulatory nutrients such as ω-3 fatty acids, nucleotides, and arginine can reduce postoperative complications after surgery for gastrointestinal cancer (46, 52). The potential mechanism may involve improved intestinal mucosal barrier function and modulation of immune responses, which reduce complications and facilitate postoperative recovery.

EIN also significantly reduced the incidence of gastrointestinal intolerance (OR = 0.44, *P* = 0.005), suggesting improved postoperative nutritional tolerance. In addition, EIN shortened the time to first flatus (MD = −3.90, *P* = 0.0002), indicating faster recovery of intestinal motility. However, no significant differences in the time to first defecation or non-infectious surgical complications such as postoperative bleeding, anastomotic leakage, or pancreatic fistula were observed, suggesting that EIN improves recovery without increasing major postoperative risks.

### 4.2 Immune, nutritional, and inflammatory indicators

EIN showed more consistent effects on humoral immunity, with postoperative increases in IgA and IgM, suggesting improved immune defense after surgery. In contrast, the effect on IgG was inconsistent and became non-significant in the sensitivity analysis. Previous meta-analyses reported improvements in humoral immunity with EIN, including IgA/IgG/IgM in Song (53) and IgG/IgM in Ying (52) and Li (55).

For cellular immunity, prior meta-analyses suggested potential improvements (e.g., CD4^+^/CD8^+^ ratio); however, conclusions regarding CD4^+^, CD8^+^, and lymphocyte-related outcomes remain conflicting across studies (52, 53, 55). In our analysis, overall effects on CD4^+^, CD8^+^, and CD4^+^/CD8^+^ were not significant, with substantial heterogeneity and generally low certainty. This inconsistency may reflect differences in immune testing time points, EIN formulations, and baseline immune status.

For nutritional biochemical markers, evidence was also unstable. Earlier studies suggested EIN increases PAB but not TRF, and effects on serum protein markers were inconsistent (52, 53, 55). In the present study, TRF and ALB levels changed after excluding heterogeneous studies, whereas no clear advantage was found in PAB, indicating that EIN alone may have limited and variable effects on nutritional markers. Postoperative nutrition indices are influenced by surgical stress, metabolism, and the duration/intensity of nutritional support, and inconsistent measurement units and sampling times further contribute to heterogeneity. Therefore, EIN should be integrated into an individualized nutrition strategy rather than relied upon alone.

Regarding inflammation, EIN was associated with lower WBC and CRP, and these findings generally persisted after sensitivity analyses, supporting a possible anti-inflammatory effect. However, results for IL-6 were inconsistent (significant only after excluding heterogeneous studies), and results for PCT and TNF-α showed no clear differences, with potential publication bias and limited number of studies. A study reported reductions in IL-6 and TNF-α with EIN; however, evidence was based on few trials (53). Overall, EIN may attenuate postoperative inflammation, but the strength of evidence remains limited and heterogeneous.

### 4.3 Sources of heterogeneity

High heterogeneity was observed in several outcomes (e.g., TC and LOS). Main sources likely include differences in EIN formulations, variations in intervention timing (preoperative, postoperative, or perioperative), and intervention duration. Moreover, inconsistent measurement units and detection time points for nutritional and inflammatory indicators across studies may have contributed to variability. Importantly, sensitivity analyses showed that the core conclusions remained stable after excluding heterogeneous studies.

### 4.4 Clinical value of subgroup analysis

Perioperative administration appears to be the most beneficial timing for EIN, as it is associated with reduced infections and total complications, shorter LOS, and improved immune indicators. In contrast, postoperative intervention mainly reduced complications without clear benefits for LOS or cellular immunity, whereas evidence for preoperative intervention remains limited because of the small number of studies. These findings suggest that early perioperative initiation of EIN better protects the intestinal mucosal barrier and support immune recovery, supporting its use during the perioperative period (53).

The benefits of EIN also appear related to intervention duration. Longer interventions ( > 10 days) were associated with shorter LOS, whereas shorter courses ( ≤ 10 days or around 7 days) still reduced postoperative complications. Therefore, EIN may prevent complications even with relatively short treatment durations, whereas extended intervention may further support recovery in patients with slower postoperative improvement. Excessively prolonged intervention may increase hospital stay and infection risk; thus, a duration of 7–10 days was recommended (15). Current ESPEN guidelines similarly recommend perioperative immunonutrition for 7–14 days in patients undergoing gastric surgery, particularly in those with moderate or severe malnutrition (10). However, substantial heterogeneity remains; thus, to optimize individualized intervention strategies, future studies should further explore patient-related factors such as nutritional status and disease severity.

Our findings sit alongside contemporary expert-consensus and systematic-review evidence. The 2021 ESPEN practical guideline on clinical nutrition in surgery recommends 5–7 days of preoperative immunonutrition for malnourished patients undergoing major upper-gastrointestinal surgery and 7–14 days of perioperative immunonutrition for patients with moderate-to-severe malnutrition, both of which are consistent with the 7–10-day window that emerged as advantageous in our subgroup analysis. The recent systematic review by Matsui et al. (Nutrients 2024) of perioperative immunonutrition in upper-gastrointestinal cancer surgery similarly reported reductions in infectious complications and length of hospital stay without a measurable mortality benefit, paralleling our results. The 2025 evidence map by Xin et al. (Clin Nutr ESPEN) likewise emphasized consistent benefits for infectious-complication endpoints alongside inconsistent effects on cellular immunity, which mirrors the divergence we observed between robust IgA/IgM increases and unstable CD4^+^/CD8^+^ effects. Taken together, our analysis supports—rather than supplants—current guideline-recommended use of perioperative EIN, particularly in nutritionally at-risk patients undergoing gastrectomy, while underscoring that several mechanistic and long-term endpoints remain insufficiently characterized.

### 4.5 Limitations

Several limitations should be considered. First, the number of studies for some outcomes was small, resulting in limited sample sizes. Second, variations in EIN formulations, intervention protocols, and outcome measurements contributed to heterogeneity. Third, most included studies had short follow-up periods, preventing evaluation of long-term outcomes such as survival or recurrence. Fourth, mortality was reported in only three trials, with heterogeneous follow-up windows and inconsistent cause-of-death adjudication, which precluded reliable quantitative pooling; future RCTs should routinely report 30-day and 1-year all-cause mortality with standardized adjudication. Fifth, nutritional assessment in the included trials relied predominantly on visceral-protein biomarkers (ALB, PAB, TRF), which are confounded by the postoperative acute-phase response; functional and composite measures such as handgrip strength, PG-SGA, and the GLIM criteria were not assessed, and we recommend GLIM-aligned reporting in future trials. Sixth, eligibility was restricted to English-language reports; although pilot screening identified no additional eligible non-English RCTs and prior methodological work suggests limited impact of English-language restriction on effect estimates in mainstream clinical topics (58), the possibility of language bias cannot be fully excluded. Seventh, Egger’s regression detected a significant small-study effect for total complications; the trim-and-fill–adjusted pooled estimate remained significant but attenuated, indicating that the magnitude of the unadjusted effect for this outcome may have been somewhat inflated by selective publication of positive small studies. Finally, the certainty of evidence as rated by GRADE was low to moderate for most outcomes (Supplementary Table 3), and the conclusions should be interpreted accordingly.

### 4.6 Clinical implications and future research directions

Overall, EIN appears to be a beneficial nutritional strategy for patients undergoing gastric cancer surgery, particularly when initiated during the perioperative period. It can reduce postoperative complications, improve gastrointestinal tolerance, and shorten hospital stay without increasing serious adverse events.

Future research should focus on large multicenter randomized controlled trials with standardized EIN formulations and intervention protocols. In addition, studies should evaluate long-term outcomes and explore factors such as baseline nutritional status and gut microbiota to better identify patients who may benefit most from EIN.

## 5 Conclusion

Compared with conventional enteral nutrition, enteral immunonutrition (EIN) is associated with reductions in postoperative infectious complications, total complications, and gastrointestinal intolerance, together with a shorter length of hospital stay and earlier return of bowel function, in patients undergoing gastric cancer surgery. Improvements were also observed in selected humoral immune (IgA, IgM) and inflammatory (WBC, CRP) markers, while effects on cellular immunity, nutritional biomarkers, IL-6, PCT, and TNF-α were inconsistent and sensitive to between-study heterogeneity. The certainty of evidence ranged from moderate for the primary endpoints to low for several secondary endpoints, and a significant small-study effect was detected for total complications. Accordingly, perioperative EIN—particularly for 7–10 days—may be considered as a component of perioperative nutritional support in selected patients, especially those with established nutritional risk, pending confirmation by adequately powered multicenter trials that include standardized mortality, GLIM-aligned nutritional assessment, and long-term oncologic endpoints.

## Data Availability

The original contributions presented in the study are included in the article/Supplementary material, further inquiries can be directed to the corresponding author.

## Footnotes

1
https://www.doi.org/10.37766/inplasy2025.12.0033

## Supplementary material

Supplementary Figure 1 Effect of enteral immunonutrition on prealbumin levels and inflammatory cytokines. **(A)** Forest plot of the primary meta-analysis, showing a significant increase in PAB levels in the EIN group. **(B)** Sensitivity analysis after exclusion of heterogeneous studies, demonstrating that the initial effect was not maintained. **(C)** Forest plot of interleukin-6 (IL-6) in the primary meta-analysis, showing no significant difference between the EIN and EN groups. **(D)** Sensitivity analysis for IL-6 after exclusion of heterogeneous studies, suggesting a reduction favoring EIN. **(E)** Forest plot of tumor necrosis factor-α (TNF-α), showing no significant difference between the EIN and EN groups.

Supplementary Figure 2 Subgroup analysis of postoperative enteral immunonutrition. **(A)** Incidence of infectious complications (IC), showing a significant reduction with postoperative EIN. **(B)** Total complication rate (TC), demonstrating a significant reduction in the EIN group. **(C)** CD4^+^ levels, showing a significant increase in patients receiving postoperative EIN. **(D)** Length of hospital stay, showing no significant difference between the EIN and EN groups. **(E)** CD8^+^ levels, showing no significant between-group difference.

Supplementary Figure 3 Subgroup analyses according to 10-day duration of enteral immunonutrition. **(A)** Total complication rate (TC) in patients receiving EIN for > 10 days, showing a significant reduction. **(B)** Length of hospital stay in patients receiving EIN for > 10 days, demonstrating a significant shortening of hospitalization. **(C)** Incidence of infectious complications (IC) in patients receiving EIN for ≤ 10 days, showing a significant reduction. **(D)** Total complication rate (TC) in patients receiving EIN for ≤ 10 days, showing a significant reduction. **(E)** Length of hospital stay in patients receiving EIN for ≤ 10 days, showing no significant difference between groups.
