# Supplementary material for: Effects of enteral immunonutrition enriched with multiple immunonutrients on clinical outcomes of patients who underwent gastric cancer surgery: a systematic review and meta-analysis
Source: Front Med (Lausanne). 2026 Jun 17;13:1844823. doi: 10.3389/fmed.2026.1844823 (PMC13318571; doi:10.3389/fmed.2026.1844823)
Supplement: Supplementary file 1 [file Table_1.docx]

**Supplementary Table 1. Egger’s Regression Test for Small-Study Effects and Trim-and-Fill Adjustment**

| **Outcome** | **No. of studies** | **Intercept (bias)** | **Std. error** | **t value** | **95% CI of intercept** | **P value** | **Interpretation** |
| --- | --- | --- | --- | --- | --- | --- | --- |
| **Overall complications** | 14 | −2.38 | 0.90 | −2.63 | −4.35 to −0.41 | 0.022 | Significant small-study effect detected |
| *Trim-and-fill (Duval & Tweedie, L0)* | 14 + 2 imputed | — | — | — | — | — | Adjusted OR = 0.46 (95% CI 0.25–0.86); 2 studies imputed on the right side of funnel; effect attenuated but remains significant |
| **Infectious complications** | 12 | −1.20 | 0.60 | −2.01 | −2.54 to 0.13 | 0.072 | Possible small-study effect; tau² = 0.0039 (low heterogeneity) |
| **Albumin level** | 11 | −0.11 | 1.74 | −0.07 | −4.06 to 3.83 | 0.949 | No small-study effect detected |

*Notes: Egger’s regression test was performed using Stata 17. The trim-and-fill procedure (Duval & Tweedie, L0 estimator) was applied to the total complications outcome, which showed a significant Egger’s test (P = 0.022). The procedure imputed 2 missing studies on the right-hand (less favorable) side of the funnel plot, producing an attenuated but still statistically significant adjusted pooled odds ratio.*Publication bias assessment was conducted using inverse-variance–weighted lnOR in Stata.

**Supplementary Table 2. Sensitivity Analysis Details: Studies Excluded, Rationale, and Effect Estimates Before and After Exclusion**

| **Outcome** | **Study(ies) excluded** | **Pooled estimate before exclusion** | **Pooled estimate after exclusion** | **Clinical/methodological reason for exclusion** | **Outcome definition across included trials** |
| --- | --- | --- | --- | --- | --- |
| **Infectious complications (IC)** | Ma C 2018 | OR = 0.48 (0.35–0.66); I² = 15% | OR = 0.57 (0.40–0.81); I² = 0% | Mixed adenocarcinoma/GIST population; zero events in EIN arm precluded stable OR estimation | Composite of SSI, pneumonia, UTI, intra-abdominal abscess, anastomotic leak with positive culture, line infection, sepsis (CDC criteria where reported) |
| **Total complications (TC)** | Fujitani K 2012; Ina S 2017; Zheng ZC 2025 | OR = 0.37 (0.19–0.71); I² = 80% | OR = 0.52 (0.37–0.73); I² = 0% | Fujitani 2012: largest trial, preoperative-only design differed from majority perioperative/postoperative protocols; Ina 2017: single-nutrient (EPA-only) formulation unlike multi-component EIN in other trials; Zheng 2025: Clavien–Dindo ≥II threshold differed from other trials’ composite endpoint | All complications reported (Clavien–Dindo ≥II where available); trial-level composite retained when breakdown unavailable |
| **Length of hospital stay (LOS)** | Liu H 2012; Wang G 2025; Farreras N 2005 | MD = −1.24 (−2.22 to −0.25); I² = 79% | MD = −0.66 (−0.96 to −0.36); I² = 0% | Wang 2025: prolonged 42-day intervention markedly longer than other protocols; Liu 2012: unusually long LOS (>20 days) in both arms reflecting institutional practice; Farreras 2005: early postoperative immunonutrition via jejunostomy, short 5-day protocol | Days from surgery to discharge (as reported) |
| **Time to first flatus** | Xu R 2020; Ma MW 2023 | MD = −6.49; P = 0.16 (I² = 93%) | MD = −3.90; P = 0.0002 (I² = 0%) | Xu 2020: hours-to-days unit inconsistency with remaining trials; Ma MW 2023: perioperative (pre+post) intervention covering 16 days, substantially longer protocol | Hours from surgery to first documented flatus |
| **IgG** | Chen DW 2005 | MD = 1.02; P = 0.03 | Effect not sustained (P > 0.05) | Different POD measurement timepoint (POD 9 vs. POD 7 in other trials); early study with limited sample size (n = 40) | Serum IgG (g/L) at specified postoperative day |
| **Transferrin (TRF)** | Chen DW 2005 | SMD = 0.34; P = 0.04 | SMD = 0.11; P = 0.35 (I² = 0%) | Measurement at POD 9 (other studies POD 5–7); small sample (n = 40) exerted disproportionate influence | Serum transferrin (g/L) at postoperative timepoint |
| **Albumin (ALB)** | Yu JJ 2024; Zheng ZC 2025 | SMD = 0.17; P = 0.24 | SMD = 0.23; P = 0.006 (I² = 0%) | Yu 2024: preoperative-only intervention; postoperative ALB reflects surgical stress rather than EIN effect. Zheng 2025: short perioperative protocol (5 days) may insufficiently influence postoperative ALB | Serum albumin (g/L) at earliest postoperative measurement |
| **Prealbumin (PAB)** | Xu R 2020; Yu JJ 2024 | SMD = 0.30; P = 0.0004 | SMD = −0.05; P = 0.62 (I² = 0%) | Xu 2020: included probiotics/fiber in EIN (eco-immunonutrition) differing from standard multi-component EIN; Yu 2024: preoperative-only protocol, PAB turnover too short to capture postoperative EIN effect | Serum prealbumin (mg/L) at postoperative timepoint |
| **CD4+** | Ma MW 2023 | SMD = 0.38; P = 0.16 (I² = 79%) | SMD = 0.83; P < 0.00001 (I² = 0%) | Measurement at postoperative month 1 (other trials POD 5–7); cells/µL vs. percentage unit inconsistency; perioperative 16-day protocol | CD4+ T cells (% or cells/µL) at postoperative timepoint |
| **WBC** | Xu R 2020; Ma MW 2023 | SMD = −0.91; P < 0.00001 (I² = 87%) | SMD = −0.43; P = 0.005 (I² = 46%) | Xu 2020: CRP/WBC values markedly higher in both arms (possible early postoperative sampling); Ma MW 2023: perioperative 16-day protocol with prolonged post-surgical measurement window | WBC count (×10⁹/L) at postoperative timepoint |
| **CRP** | Yu JJ 2024 | SMD = −1.43; P < 0.00001 (I² = 89%) | SMD = −0.86; P < 0.0001 (I² = 29%) | Preoperative-only intervention; CRP measured at POD 3 (other studies POD 5–7); median/IQR values converted to mean/SD, introducing imprecision | Serum CRP (mg/L) at postoperative timepoint |
| **IL-6** | Yu JJ 2024 | SMD = −0.15; P = 0.74 (I² = 81%) | SMD = −0.80; P = 0.001 (I² = 0%) | Preoperative-only EIN; median/IQR-to-mean/SD conversion; markedly higher baseline IL-6 than other cohorts | Serum IL-6 (pg/mL) at postoperative timepoint |

*Abbreviations: OR, odds ratio; MD, mean difference; SMD, standardized mean difference; CI, confidence interval; I², heterogeneity statistic; SSI, surgical site infection; UTI, urinary tract infection; POD, postoperative day; EIN, enteral immunonutrition; GIST, gastrointestinal stromal tumor; NS, not significant.*

**Supplementary Table 3. GRADE Summary of Findings: Certainty of Evidence for Each Outcome**

| **Outcome** | **No. of studies** | **Risk of bias** | **Inconsistency** | **Indirectness** | **Imprecision** | **Publication bias** | **Overall certainty** | **Effect estimate (95% CI)** | **Absolute effect per 1000** | **Summary** |
| --- | --- | --- | --- | --- | --- | --- | --- | --- | --- | --- |
| **Infectious complications** | 12 | Not serious | Not serious (I² = 15%) | Not serious | Not serious | Undetected (Egger P = 0.072) | ⊕⊕⊕⊖ Moderate | OR 0.48 (0.35–0.66) | 122 fewer per 1000 (from 80 to 153 fewer) | Downgraded one level for some concerns about blinding in open-label trials |
| **Total complications** | 14 | Not serious | Serious (I² = 80%) | Not serious | Not serious | Detected (Egger P = 0.022); trim-and-fill adjusted OR = 0.46 | ⊕⊕⊖⊖ Low | OR 0.37 (0.19–0.71) | 175 fewer per 1000 (from 78 to 243 fewer) | Downgraded one level for inconsistency; one level for publication bias |
| **Length of hospital stay** | 9 | Not serious | Serious (I² = 79%) | Not serious | Not serious | Undetected | ⊕⊕⊕⊖ Moderate | MD −1.24 days (−2.22 to −0.25) | — | Downgraded one level for inconsistency |
| **GI intolerance** | 6 | Not serious | Not serious (I² = 0%) | Not serious | Not serious | Undetected | ⊕⊕⊕⊖ Moderate | OR 0.44 (0.25–0.78) | 90 fewer per 1000 (from 35 to 126 fewer) | Downgraded one level for some concerns about blinding |
| **Time to first flatus** | 4 | Not serious | Serious (I² = 93%) | Not serious | Serious | Not assessable (k < 10) | ⊕⊖⊖⊖ Very low | MD −6.49 h (NS in primary analysis) | — | Downgraded for inconsistency, imprecision, few studies |
| **IgA** | 3 | Not serious | Not serious | Not serious | Not serious | Not assessable (k < 10) | ⊕⊕⊕⊖ Moderate | MD 0.38 (0.25–0.51) | — | Downgraded one level for small total sample size |
| **IgM** | 4 | Not serious | Not serious | Not serious | Not serious | Undetected | ⊕⊕⊕⊖ Moderate | MD 0.31 (0.13–0.49) | — | Downgraded one level for some concerns about blinding |
| **IgG** | 4 | Not serious | Serious | Not serious | Serious | Not assessable (k < 10) | ⊕⊖⊖⊖ Very low | MD 1.02 (P = 0.03); unstable after sensitivity analysis | — | Downgraded for inconsistency and imprecision; effect not robust |
| **Albumin (ALB)** | 11 | Not serious | Serious (I² = 63%) | Not serious | Serious | Undetected (Egger P = 0.949) | ⊕⊖⊖⊖ Very low | SMD 0.17 (−0.12 to 0.46); NS | — | Downgraded for inconsistency and imprecision; unstable result |
| **Prealbumin (PAB)** | 5 | Not serious | Serious | Not serious | Not serious | Not assessable (k < 10) | ⊕⊕⊖⊖ Low | SMD 0.30 (P = 0.0004); not maintained after sensitivity | — | Downgraded for inconsistency; effect not robust |
| **Transferrin (TRF)** | 4 | Not serious | Serious | Not serious | Serious | Not assessable (k < 10) | ⊕⊖⊖⊖ Very low | SMD 0.34 (P = 0.04); not maintained after sensitivity | — | Downgraded for inconsistency and imprecision |
| **CD4+** | 5 | Not serious | Serious (I² = 79%) | Serious | Not serious | Not assessable (k < 10) | ⊕⊖⊖⊖ Very low | SMD 0.38 (P = 0.16; NS) | — | Downgraded for inconsistency and indirectness (unit heterogeneity) |
| **CD8+** | 5 | Not serious | Serious | Serious | Serious | Not assessable (k < 10) | ⊕⊖⊖⊖ Very low | SMD −0.28 (NS) | — | Downgraded for inconsistency, indirectness, imprecision |
| **WBC** | 6 | Not serious | Serious (I² = 87%) | Not serious | Not serious | Not assessable (k < 10) | ⊕⊕⊖⊖ Low | SMD −0.91 (P < 0.00001) | — | Downgraded for inconsistency; effect persisted after sensitivity analysis |
| **CRP** | 4 | Not serious | Serious (I² = 89%) | Not serious | Serious | Not assessable (k < 10) | ⊕⊖⊖⊖ Very low | SMD −1.43 (P < 0.00001) | — | Downgraded for inconsistency and imprecision |
| **IL-6** | 4 | Not serious | Serious (I² = 81%) | Not serious | Serious | Not assessable (k < 10) | ⊕⊖⊖⊖ Very low | SMD −0.15 (P = 0.74; NS) | — | Downgraded for inconsistency and imprecision |
| **TNF-α** | 3 | Not serious | Serious | Not serious | Serious | Not assessable (k < 10) | ⊕⊖⊖⊖ Very low | SMD (P = 0.19; NS) | — | Downgraded for inconsistency, imprecision, and few studies |
| **PCT** | 2 | Not serious | Not serious | Not serious | Very serious | Not assessable (k < 10) | ⊕⊖⊖⊖ Very low | P = 0.98 (NS) | — | Downgraded two levels for very serious imprecision (n = 184, 2 trials) |

*GRADE certainty ratings: ⊕⊕⊕⊕ High; ⊕⊕⊕⊖ Moderate; ⊕⊕⊖⊖ Low; ⊕⊖⊖⊖ Very low. Downgrading domains: risk of bias, inconsistency, indirectness, imprecision, publication bias. Absolute effects calculated for dichotomous outcomes assuming control-group risk from the meta-analysis.*

*Abbreviations: OR, odds ratio; MD, mean difference; SMD, standardized mean difference; CI, confidence interval; GI, gastrointestinal; N*
