# Supplementary figures and images for: Effects of enteral immunonutrition enriched with multiple immunonutrients on clinical outcomes of patients who underwent gastric cancer surgery: a systematic review and meta-analysis
Source: Front Med (Lausanne). 2026 Jun 17;13:1844823. doi: 10.3389/fmed.2026.1844823 (PMC13318571; doi:10.3389/fmed.2026.1844823)

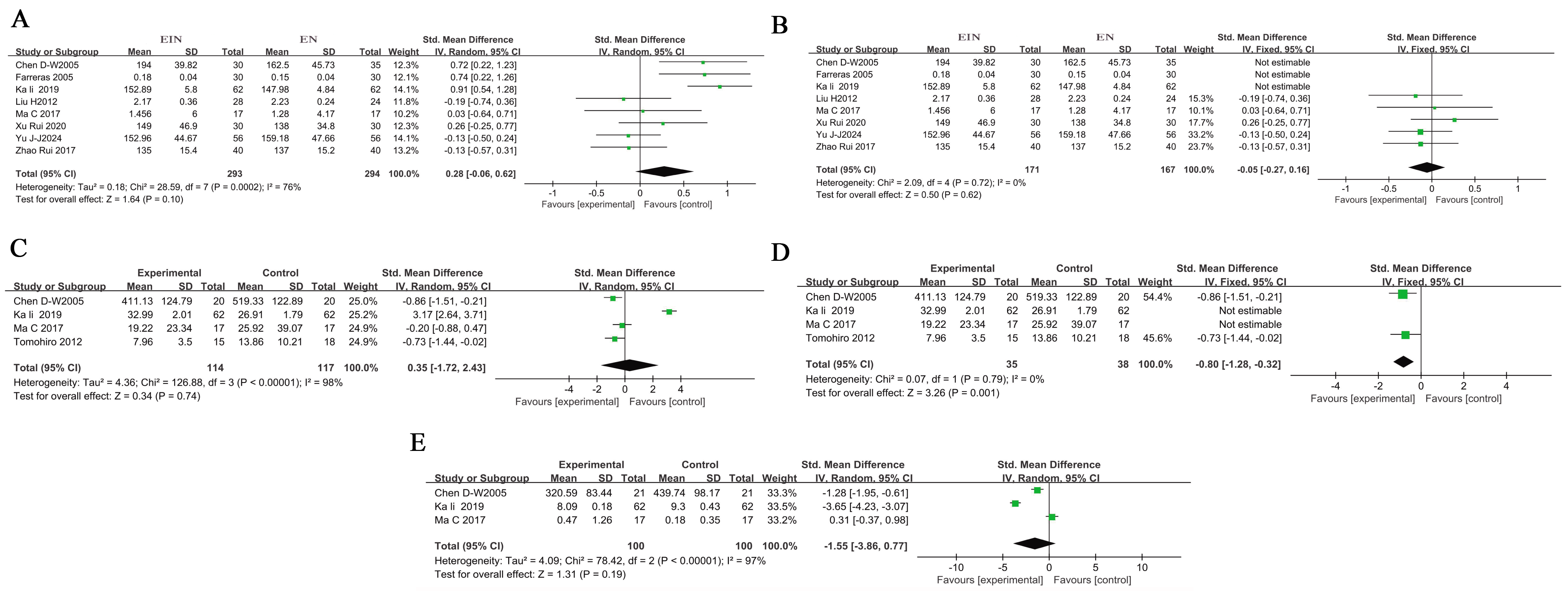

Supplement: Supplementary Figure 1 — Effect of enteral immunonutrition on prealbumin levels and inflammatory cytokines. (A) Forest plot of the primary meta-analysis, showing a significant increase in PAB levels in the EIN group. (B) Sensitivity analysis after exclusion of heterogeneous studies, demonstrating that the initial effect was not maintained. (C) Forest plot of interleukin-6 (IL-6) in the primary meta-analysis, showing no significant difference between the EIN and EN groups. (D) Sensitivity analysis for IL-6 after exclusion of heterogeneous studies, suggesting a reduction favoring EIN. (E) Forest plot of tumor necrosis factor-α (TNF-α), showing no significant difference between the EIN and EN groups. [file Image_1.tif]

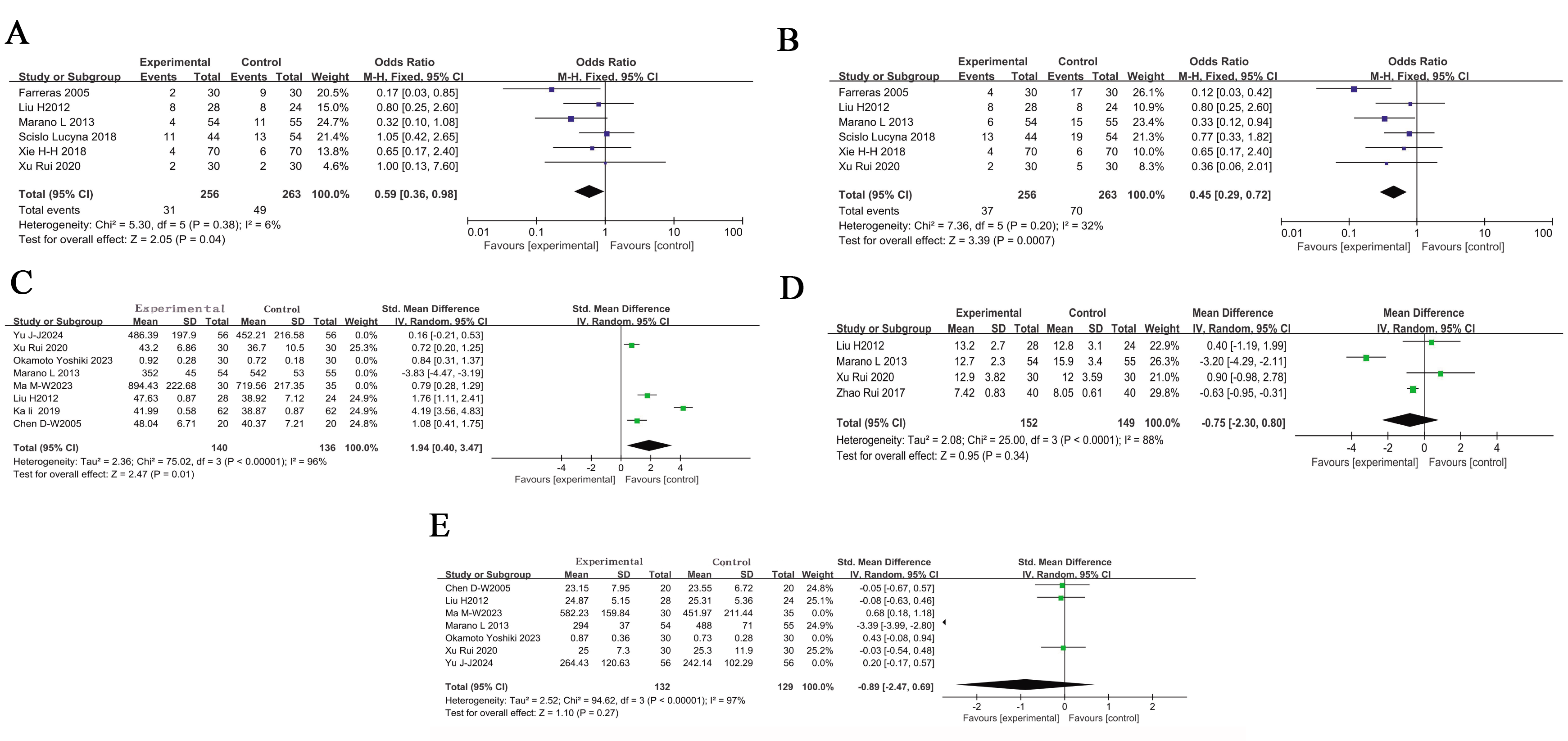

Supplement: Supplementary Figure 2 — Subgroup analysis of postoperative enteral immunonutrition. (A) Incidence of infectious complications (IC), showing a significant reduction with postoperative EIN. (B) Total complication rate (TC), demonstrating a significant reduction in the EIN group. (C) CD4+ levels, showing a significant increase in patients receiving postoperative EIN. (D) Length of hospital stay, showing no significant difference between the EIN and EN groups. (E) CD8+ levels, showing no significant between-group difference. [file Image_2.tif]

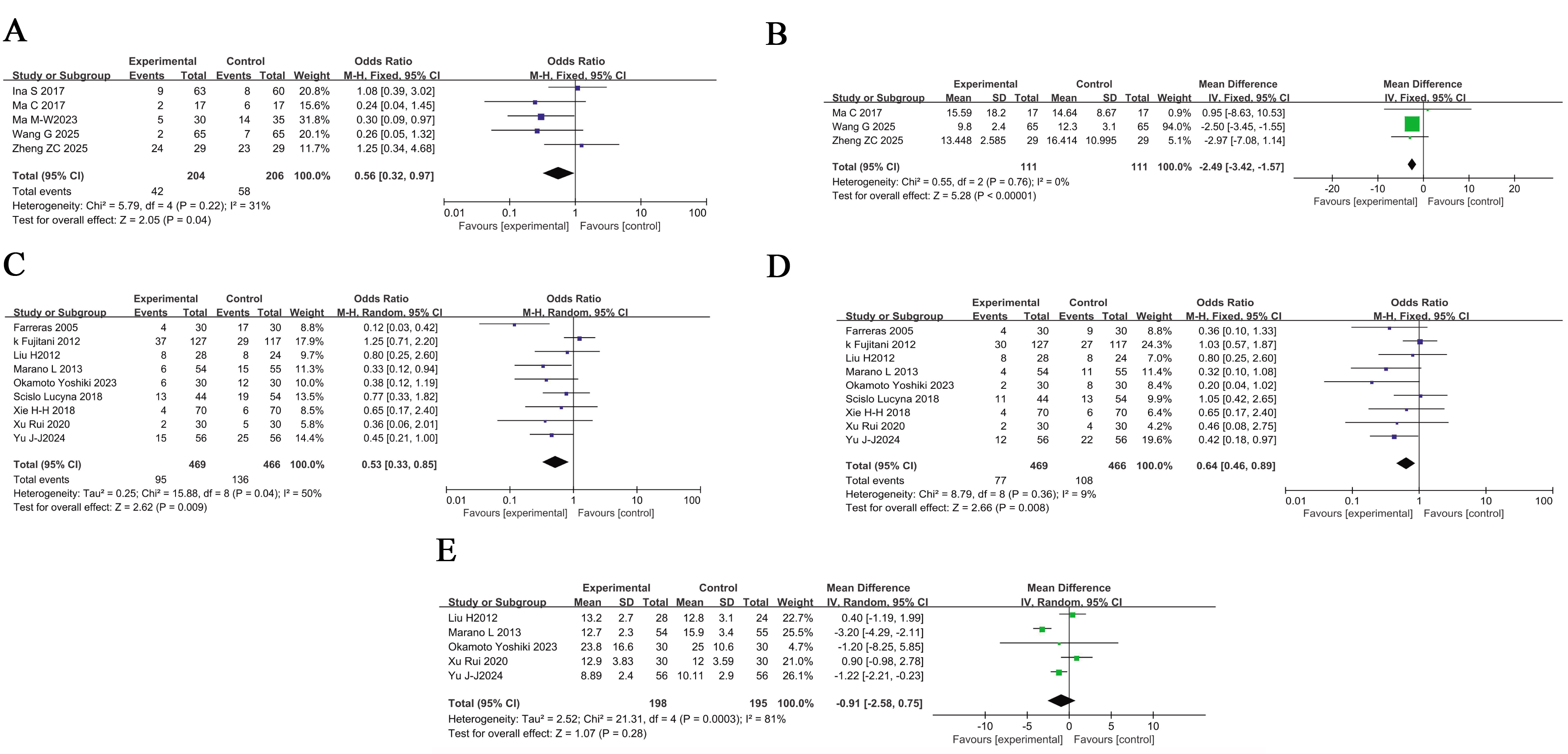

Supplement: Supplementary Figure 3 — Subgroup analyses according to 10-day duration of enteral immunonutrition. (A) Total complication rate (TC) in patients receiving EIN for > 10 days, showing a significant reduction. (B) Length of hospital stay in patients receiving EIN for > 10 days, demonstrating a significant shortening of hospitalization. (C) Incidence of infectious complications (IC) in patients receiving EIN for ≤ 10 days, showing a significant reduction. (D) Total complication rate (TC) in patients receiving EIN for ≤ 10 days, showing a significant reduction. (E) Length of hospital stay in patients receiving EIN for ≤ 10 days, showing no significant difference between groups. [file Image_3.tif]
